# The bacterial tRNA-modifying enzyme tRNA^Ile^ lysidine synthetase is genetically conserved but catalytically variable

**DOI:** 10.1016/j.jbc.2025.110688

**Published:** 2025-09-04

**Authors:** Marc J. Muraski, Ferdiemar C. Guinto, Emil M. Nilsson, Jorge B. Dabdoub, Samantha C. Robinson, Yiyang Jiang, Zhen Shen, Rebecca W. Alexander

**Affiliations:** 1 Department of Chemistry and Center for Molecular Signaling, Wake Forest University, Winston-Salem, North Carolina, USA; 2 Pharmaceutical Services Group, ThermoFisher Scientific, High Point, North Carolina, USA; 3 Department of Chemistry, University of California Davis, Davis, California, USA

**Keywords:** enzyme kinetics, tRNA, mutagenesis, translation, TilS, evolution, RNA modification

## Abstract

The AUA isoleucine codon is generally rare and used with varying frequency in bacterial genomes. The tRNA^Ile2^ responsible for decoding this trinucleotide must be modified at the wobble position by tRNA^Ile^ lysidine synthetase (TilS) prior to aminoacylation and accommodation at the ribosome. To test the hypothesis that TilS catalytic efficiency correlates with AUA frequency, we cloned *tilS* genes from bacteria with varying AUA codon usage. A previous study identified mutations in the *Burkholderia cenocepacia tilS* gene affecting locations distal to the catalytic domain that caused large fitness increases by enabling earlier exponential growth in minimal growth media. We made comparable mutations in TilS orthologs to better understand the effect of amino acid substitutions at these locations. While the *tilS* gene is present in nearly all bacteria, orthologs exhibit notable differences in substrate recognition and catalytic efficiency that are not readily correlated with codon usage.

Translation of a protein from its genetically encoded template requires dozens of biomolecular binding events and chemical reactions. Even discounting DNA polymorphisms, the sets of proteins present in otherwise identical cells vary in type and amount depending on both chance encounters and enzymatic efficiency of these individual steps (1). Organisms have evolved mechanisms to limit the intrinsic noise of translation and efficiently assemble the proteins needed for cellular function. One critical step in faithful translation of the genetic code is accurate attachment of amino acids to tRNA adaptor molecules (2).

The tRNA trinucleotide anticodon typically serves as a dominant identity element for tRNA recognition and subsequent aminoacylation by a cognate aminoacyl-tRNA synthetase (AARS) (2, 3). The anticodon also provides specificity during ribosomal decoding through direct base pairing between the tRNA anticodon and the mRNA codon. Given its dual role in aminoacylation and decoding, the anticodon is considered unique to a given amino acid and a critical contributor to translational accuracy. One exception is the CAU anticodon in most bacteria and archaea (4, 5). The CAU anticodon is a dominant identity element for methionyl-tRNA synthetase (MetRS) across all domains of life and decodes the single methionine-specific AUG codon (6). The GAU anticodon of isoleucine-specific tRNAs decodes both the AUC and AUU codons, the latter through wobble-pairing, whereas most bacteria and archaea lack a tRNA to directly read the AUA isoleucine codon. Instead, a minor isoleucine tRNA acceptor (tRNA^Ile2^) is, like tRNA^Met^, initially transcribed with a CAU anticodon. Translational ambiguity would result if tRNA^Ile2^ retained the CAU anticodon, but the C34 wobble position is post-transcriptionally modified to lysidine in bacteria by tRNA^Ile^ lysidine synthetase (TilS) (7, 8, 9). In a parallel adaptation, archaea use the enzyme tRNA^Ile^ agmatidine synthetase to install agmatidine at C34 (10). Despite the different wobble modifications used by eubacteria and archaea, recent cryo–electron microscopy structures reveal similar decoding strategies at the ribosome (11, 12). The newly identified tRNA^Ile2^ wobble modification aminovaleramide, found in a subset of bacteria and some plant organelles, is a derivative of lysidine and selectively decodes the AUA codon in a structurally similar approach to lysidine and agmatidine (13). In each case, the N34 2-amino group makes a single hydrogen bond to the adenine N1 at the third position of the AUA codon, whereas the N34 extended tail expands the interaction by H-bonding to the 2′-OH of the next nucleotide in the sequence.

The *tilS* gene is highly conserved across the bacterial domain, and unsuccessful attempts to delete it in the model organisms *Escherichia coli* and *Bacillus subtilis* suggest it is an essential bacterial gene (8, 9, 14, 15). The TilS enzyme is unique to bacteria and highly conserved; as such, it has been proposed as an attractive antimicrobial drug target (16).

tRNA^Met^ and pre-tRNA^Ile2^ are both initially transcribed with the CAU anticodon, although they are destined for two distinct fates. Given that AUA is a minor or even rare codon in most organisms and that tRNA abundance correlates with codon usage (17, 18), it is likely that more of the CAU-containing tRNAs are destined to decode the methionine-specific AUG codon. Nevertheless, some will ultimately decode the isoleucine-specific AUA codon, and TilS serves to differentiate pre-tRNA^Ile2^ from tRNA^Met^. This suggests that MetRS and TilS enzymes must efficiently discriminate between two CAU-containing tRNAs to faithfully translate the genetic code. While the CAU anticodon has long been seen as a dominant identity element for MetRS, we have previously demonstrated that MetRS orthologs distinguish between tRNA^Met^ and unmodified tRNA^Ile2^ to varying degrees (19). At least some MetRS enzymes use structural and or sequence features of tRNA^Met^ outside the anticodon to reject the CAU-containing near-cognate tRNA^Ile2^. In a parallel fashion, TilS efficiency also depends on tRNA^Ile2^ nucleotides outside the anticodon, as has been shown by us and others (20, 21).

We recently showed that strains of *Burkholderia cenocepacia* containing single nucleotide changes in the *tilS* gene causing nonsynonymous amino acid substitutions had increased fitness compared with the wildtype strain in a specific minimal media (22). This cellular fitness increase occurred despite modest losses of TilS affinity for the tRNA^Ile2^ substrate *in vitro* and reduced enzymatic function to produce lysidine *in vivo*. We wondered whether lysidinylation of tRNA^Ile2^ might serve as a metabolic signal in *B*. *cenocepacia* and whether TilS activity correlates with AUA frequency across bacterial species. Here, we examine the catalytic activities of selected TilS enzymes and investigate whether the loss of function observed in fitness-enhancing variants of *B*. *cenocepacia* TilS (BcTilS) is recapitulated in orthologous enzymes.

## Results

### BcTilS uses different tRNA identity elements than *Escherichia coli* TilS

**Figure 1:**
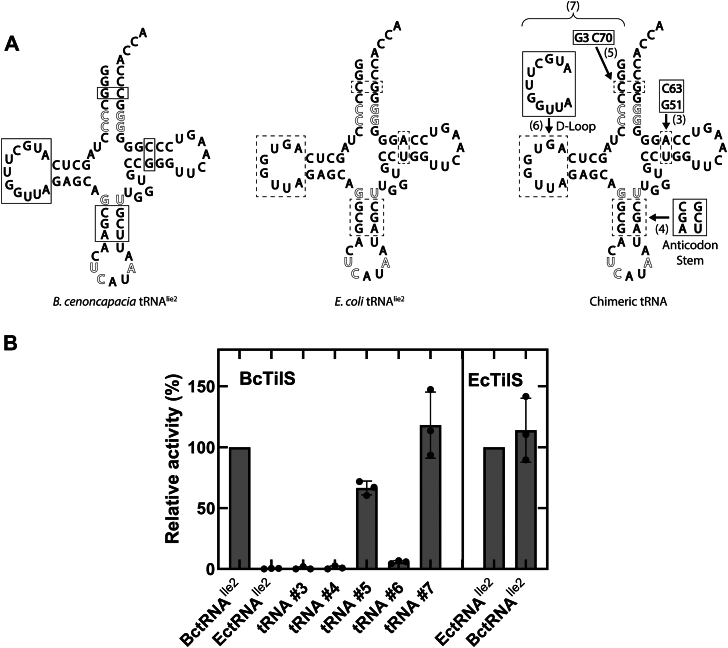
**tRNA^Ile2^ identity elements vary between bacteria**. *A*, BctRNA^Ile2^ and EctRNA^Ile2^ cloverleaf representations; *outlined nucleotides* are established identity elements for EcTilS and conserved in both EctRNA^Ile2^ and BctRNA^Ile2^ (20). Five chimeric tRNA transcripts were synthesized. *Solid outlined boxes* indicate elements of BctRNA^Ile2^ transposed into EctRNA^Ile2^ in areas marked by *dashed outlined boxes*. tRNAs 3 through 6 introduced substitutions of single base pairs or motifs. tRNA 7 combines the substitutions introduced in tRNAs 5 and 6. *B*, relative activity of BcTilS (500 nM) and EcTilS (50 nM) for BctRNA^Ile2^, EctRNA^Ile2^, and chimeric transcripts (10 μM) based on the schematic in *A*. Error bars represent the standard deviation of three technical replicates. BctRNA^Ile2^, *Burkholderia cenocepacia* tRNA^Ile2^; EcTilS, *Escherichia coli* TilS; EctRNA^Ile2^, *Escherichia coli* tRNA^Ile2^.

The *tilS* gene is considered essential, given its conservation in ∼98% of annotated bacterial genomes and the observation that its deletion in *E*. *coli* or *B*. *subtilis* results in a lethal phenotype (14, 15). We recently demonstrated that mutations in BcTilS displayed a growth benefit in 1% galactose minimal media despite a reduction in the lysidinylation functionality of TilS; the source of this benefit was determined to be a 3.5-h reduction in lag phase growth (22). To further understand BcTilS function, we initiated a comparative study with its ortholog from the Gram-negative model organism *Escherichia coli* TilS (EcTilS). Mutagenic analysis of the EcTilS–tRNA system was previously carried out by Ikeuchi *et al*. (20), demonstrating that the C4:G69 and C5:G68 base pairs in the acceptor stem of its substrate tRNA^Ile2^ are direct contact sites for EcTilS and serve as dominant identity elements for lysidinylation. Transcript tRNA^Ile2^ is an efficient substrate for EcTilS *in vitro* (20); therefore, all kinetic analyses described later were carried out using *in vitro* transcribed tRNA. The previously determined EcTilS identity elements are conserved in *B*. *cenocepacia* tRNA^Ile2^ (BctRNA^Ile2^) (Fig. 1*A*), and indeed, this orthologous tRNA was an efficient substrate for EcTilS in an *in vitro* lysidinylation assay (Fig. 1*B*, *right panel*). In contrast, BcTilS was unable to use *Escherichia coli* tRNA^Ile2^ (EctRNA^Ile2^) as a substrate (Fig. 1*B*, *left panel*). This result suggests that BcTilS requires additional or alternate identity elements compared with EcTilS, or that EctRNA^Ile2^ has sequence or structural features that serve as antideterminants of BcTilS. To better understand how BcTilS recognizes its tRNA substrate, we used a gain-of-function approach. Building on an EctRNA^Ile2^ scaffold, BctRNA^Ile2^ elements were sequentially added, and the lysidinylation capacity of each chimeric tRNA was tested.

First, a G51:C63 base pair in the TΨC-stem (chimeric tRNA 3) and then an anticodon stem chimera (tRNA 4) were independently synthesized and evaluated for lysidine formation. Neither change enhanced lysidinylation by BcTilS relative to wildtype EctRNA^Ile2^ (Fig. 1*B*). Next, the C3:G70 base pair of EctRNA^Ile2^ was exchanged for the corresponding G3:C70 BctRNA^Ile2^ base pair (tRNA 5). This transversion recovered 60% of the cognate BctRNA^Ile2^ lysidinylation. Substitution of the EctRNA^Ile2^ D-loop with the BctRNA^Ile2^ D-loop (tRNA 6) restored 6% lysidinylation activity. The combination of the G3:C70 transversion and the D-loop substitution (tRNA 7) generated a chimeric tRNA substrate that was lysidinylated by BcTilS at a level indistinguishable from that of the native BctRNA^Ile2^.

### Genetic distribution representation

While investigating the BctRNA^Ile2^ identity elements, we noted that the initial rate for lysidine formation was ∼24-fold higher for EcTilS compared with BcTilS (139 nM/min *versus* 5.8 nM/min, Table S1). We considered that AUA codon usage might influence the enzymatic efficiency of TilS. The AUA codon is even more rare (0.6 per 1000 codons) in the GC-rich *B*. *cenocepacia* than in *E*. *coli* (4.9 per 1000 codons). We have previously investigated the ability of MetRS orthologs to aminoacylate the near-cognate tRNA substrate tRNA^Ile2^, which is the lysidinylation target of TilS (19). We predicted that TilS is more efficient in species where MetRS discriminates less effectively against tRNA^Ile2^. To connect the current study of TilS with the broader question of bacterial translation specificity, we cloned *tilS* genes from organisms previously investigated: *E*. *coli*, *Borrelia burgdorferi*, *Bacteroides fragilis*, *Helicobacter pylori*, *Mycoplasma penetrans*, *Mycobacterium smegmatis*, and *Staphylococcus pneumoniae*. The *Pseudomonas aeruginosa* and *Geobacillus kaustaphilus* TilS (GkTilS) enzymes were added to the library of orthologs not originally reported. *Pseudomonas aeruginosa* TilS (PaTilS) was chosen because of the pathogenic parallels between *P*. *aeruginosa* and *B*. *cenocepacia*, whereas GkTilS is the only enzyme whose tRNA-bound structure has been solved to date. These orthologs represent varying GC content, bacterial clades, and TilS classifications (Table 1). Type I TilS enzymes have 2 C-terminal domains (CTD1 and CTD2) connected to the N-terminal lysidinylation active site by an α-helical linker. The CTD2 domain has been shown to contact nucleotides in the acceptor stem of tRNA^Ile2^ (23), and we have demonstrated that residues in a helix–turn–helix motif of CTD2 are essential for lysidinylation despite the ∼60 Å separation from the active site (21). Type II enzymes lack the CTD2 domain; we estimate that about 30% of TilS enzymes are in the type II category.

**Table 1:** Kinetic parameters of TilS orthologs

Organism	TilS type	AUA usage	Species GC %	pH optimum	*K*_*M*_^a^ (μM)	*k*_obs_^a^ (min^-1^)	*k*_obs_/*K*_*M*_ (μM^-1^ min^-1^)	*K*_*d*_^a^ (μM)
*B*. *cenocepacia*	I	0.6	67.4	9.0	7.7 ± 2.8	0.03 ± 0.01	0.004 ± 0.001	1.5 ± 0.4
*E*. *coli*	I	4.9	51.8	9.0	0.8 ± 0.2	4.2 ± 1.9	5.0 ± 1.3	1.5 ± 0.4
*G*. *kaustaphilus*	I	2.9	52.8	9.0	0.12 ± 0.02	0.67 ± 0.11	5.55 ± 0.08	0.7 ± 0.1
*B*. *fragilis*	I	16.8	44.1	8.5	5.2 ± 1.2	1.6 ± 0.2	0.32 ± 0.09	0.8 ± 0.1
*P*. *aeruginosa*	I	0.9	67.1	9.0	1.1 ± 0.4	0.32 ± 0.03	0.3 ± 0.1	1.3 ± 0.3
*M*. *smegmatis*	II	0.9	67.8	8.5	1.7 ± 0.6	0.08 ± 0.01	0.05 ± 0.01	5.1 ± 0.8
*H*. *pylori*	II	9.0	39.8	8.5	0.6 ± 0.2	0.55 ± 0.05	1.0 ± 0.3	0.9 ± 0.1

a Average of three trials ± standard deviation; *K*_*M*_ and *K*_*d*_ values are reported for tRNA^Ile2^.

**Figure 2:**
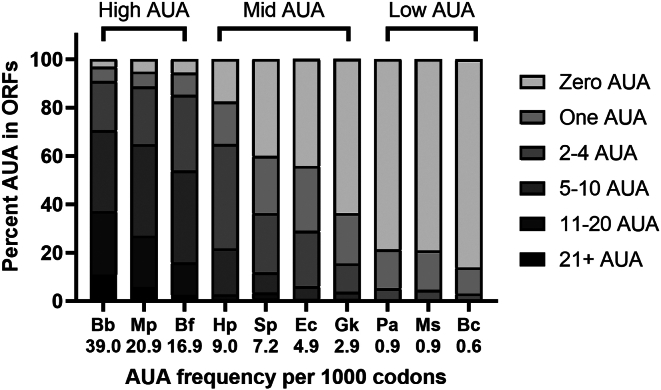
**Classification of AUA codon distribution in select organisms**. Coding sequences of 10 genomes were analyzed for AUA codon distribution in ORFs. Genes were grouped into 21+, 11 to 20, 5 to 10, 2 to 4, 1, and 0 AUA codons per ORF, with the percentage of genes in each group stacked. The overall frequency of AUA codon use is listed below each species abbreviation on the *X*-axis.

The number of AUA per 1000 codons in a particular bacterial species does not fully represent whether some genes with multiple AUA codons might be disproportionately dependent on TilS for expression. To begin comparing TilS function with AUA use, we analyzed the genomic distribution of AUA codons in protein-coding genes for *B*. *cenocepacia* and the 9 bacterial species listed previously (Fig. 2). We grouped genomes into three categories of AUA usage: low (<2 per 1000 codons), medium (∼3–10 per 1000), and high (>10 per 1000). There is a wide range of AUA usage across bacteria, including those species represented here: *B*. *cenocepacia* is at the very low end, with 0.6 AUA per 1000 codons and 86% of genes lacking even a single AUA codon. *E*. *coli* is in the middle of the species compared with 4.9 AUA codons per 1000; 55% of *E*. *coli* genes have at least one AUA, and 29% have two or more. At the upper end is *B*. *burgdorferi*, with 39 AUA codons per 1000 and 10% of genes containing 21 or more AUAs. We further examined AUA codon usage in the 40 most highly expressed bacterial genes (HEGs), consisting primarily of ribosomal proteins and translational factors (24). The frequency of AUA usage in HEGs is reduced for all the species compared here, with a greater decrease for species already low in genomic AUA abundance. In *P*. *aeruginosa*, *M*. *smegmatis*, and *B*. *cenocepacia*, where AUA is already used less than once per 1000 codons, AUA is not found at all in HEGs (Table S4). The TilS homologs from these species exhibited among the lowest lysidinylation activities *in vitro*. Interestingly, the occurrence of isoleucine from all three codons (AUC, AUU, and AUA) is also variable across these species but is inverse of the AUA case. Isoleucine incorporation is greater in HEGs relative to genomic coding sequences for species that use AUA sparingly. A rationale for such codon switching is not immediately obvious and requires further investigation.

### Kinetic characterization of TilS orthologs

To explore the range of lysidinylation efficiency in these organisms, we examined kinetic parameters of TilS enzymes with their cognate tRNA^Ile2^ substrates. The BcTilS enzyme represents the least efficient example, with a *k*_obs_/*K*_*M*_ more than 1000-fold lower than the most efficient EcTilS and GkTilS enzymes (Table 1). The defect in BcTilS catalysis is in both the affinity for tRNA^Ile2^ (*K*_*M*_) and the catalytic step (*k*_obs_). Other orthologs range from 50-fold to only 1.5-fold less efficient than the model EcTilS, with both *k*_obs_ and *K*_*M*_ contributions.

We also investigated how tightly each TilS enzyme bound its tRNA^Ile2^ substrate using a gel shift assay with radiolabeled tRNA to determine the *K*_*d*_ for each enzyme (Fig. S1). Orthologs exhibited similar tRNA affinities (ranging from 0.7 to 1.5 μM), except for *M*. *smegmatis* TilS (MsTilS) (5.1 μM) (Table 1). The GkTilS–tRNA^Ile2^ and *Helicobacter pylori* TilS (HpTilS)–tRNA^Ile2^ complexes became insoluble when exceeding 10 μM TilS.

### Varying importance of distal residues to TilS activity

**Figure 3:**
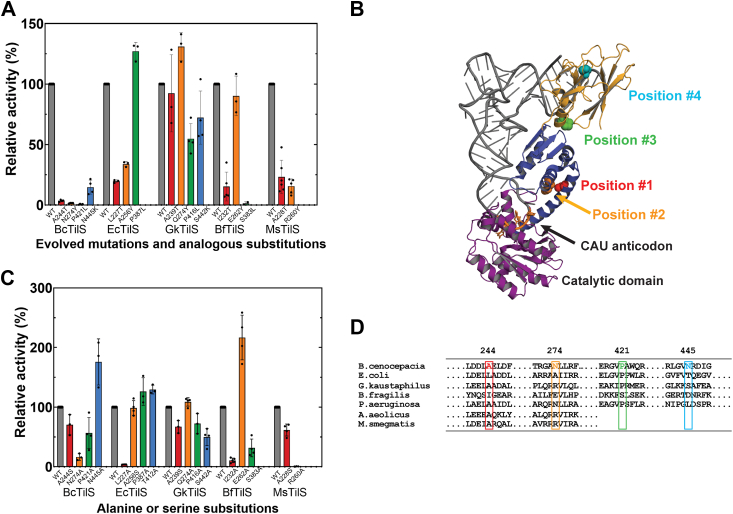
**TilS substitutions exhibit variable impact on catalytic activity across species**. The relative initial rate of each TilS variant compared with its wildtype counterpart was determined through a lysidinylation assay. The positions are based on the identified evolved mutations in BcTilS, which are as follows: position #1 (A244T, *red*), position #2 (N274Y, *orange*), position #3 (P421L, *green*), and position #4 (N445K, *blue*). Error bars represent the standard deviation of three technical replicates. *A*, relative rates for the analogous evolved mutations; *B*, crystal structure of the *Geobacillus kaustaphilus* TilS in complex with *Bacillus subtilis* tRNA^Ile2^; the CAU anticodon is highlighted as *orange licorice*, and the mutational sites are shown as space-filling side chains corresponding to the colors in other *panels*. The N-terminal catalytic domain is shown in *purple*, CTD1 in *blue*, and CTD2 in *gold* (3A2K) (23). *C*, relative rates for the alanine or serine variants of positions corresponding to evolved BcTilS mutations; *D*, sequence alignment of TilS orthologs used in mutational studies with *G*. *kaustaphilus* and *Mycobacterium smegmatis* serving as type I and type II representative sequences. BcTilS, *Burkholderia cenocepacia* TilS.

Previously, we showed that four nonsynonymous substitutions in BcTilS selected during experimental evolution decreased lysidinylation by 75% or more (Fig. 3*A*) (22). Each position was distant from the lysidinylation active site (ranging from 27 to 76 Å away, based on the GkTilS crystal structure, Fig. 3*B* (23)). We concluded that these residues, which vary in their phylogenetic conservation, contribute to tRNA recognition and/or dynamic signaling to the BcTilS catalytic core. To evaluate the contributions of these positions to enzyme function in other orthologs, we generated corresponding substitutions in EcTilS, GkTilS, *Bacteroides fragilis* TilS (BfTilS), and MsTilS (Fig. 3, *A* and *D*; Fig. S3). We also generated alanine substitutions at each of these positions, or if the position was already an alanine, a serine residue was introduced. Many substitutions made to recapitulate evolved BcTilS SNPs (Table S1) resulted in decreased activity; however, the magnitude of loss was not as great as for the BcTilS variants (Fig. 3*A*). In general, the GkTilS ortholog was functionally robust, with each variant exhibiting at least 50% activity compared with the wildtype enzyme. Most alanine substitutions for each ortholog (Table S2) retained more activity relative to each wildtype enzyme than did the substitutions mimicking evolved mutations (Fig. 3*C*). Substitutions at position #4 near the protein’s C terminus produced insoluble EcTilS for the evolved variant (T412K), and BfTilS was insoluble for both the evolved (D412K) and alanine (D412A) variants. Type II MsTilS lacks the extended CTD (CTD2) found in type I TilS enzymes, so substitutions at positions 3 and 4 could not be incorporated into this enzyme. *A*quifex *aeolicus* is also a type II TilS, as indicated by its truncated sequence (Fig. 3*D*).

### Discrimination of near-cognate substrates by MetRS

**Figure 4:**
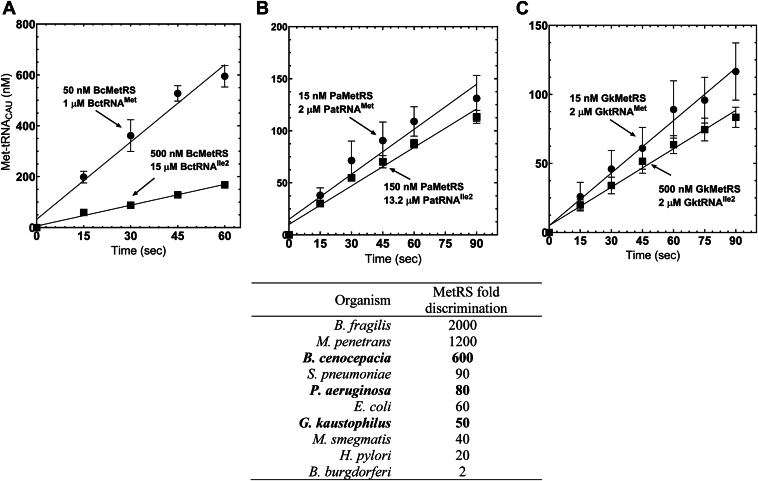
**MetRS discrimination of near-cognate tRNA^Ile2^**. MetRS enzymes from *Burkholderia cenocepacia* (BcMetRS), *Pseudomonas aeruginosa* (PaMetRS), and *Geobacillus kaustophilus* (GkMetRS) were evaluated for their capacity to methionylate the near cognate ${\text{tRNA}}_{\text{CAU}}^{\mathrm{Ile}2}$. *A*, 50 nM BcMetRS was used with 1 μM BctRNA^Met^ or 500 nM BcMetRS with 15 μM BctRNA^Ile2^. *B*, 15 nM PaMetRS was used with 2 μM PatRNA^Met^ or 150 nM PaMetRS with 13.2 μM PatRNA^Ile2^. *C*, 15 nM GkMetRS was used with 2 μM GktRNA^Met^ or 500 nM GkMetRS with 2 μM GktRNA^Ile2^. Bold entries in the summary table are from this work; nonbold entries are from the study of Jones *et al*. (19).

It has previously been reported that unmodified tRNA^Ile2^ and tRNA^Met^, which share a CAU anticodon motif, are both able to be aminoacylated by MetRS and could potentially decode the AUG codon during translation (9, 20, 25, 26, 27). The phenomenon was initially observed in *E*. *coli*, wherein in the absence of lysidine modification, tRNA^Ile2^ was a viable substrate for MetRS *in vitro* (26). However, previously we showed that MetRS aminoacylation of near-cognate tRNA^Ile2^ varies across species (19). To test whether unmodified tRNA^Ile2^ would be an efficient substrate for *B*. *cenocepacia* MetRS (BcMetRS), *P*. *aeruginosa* MetRS (PaMetRS), and *G*. *kaustaphilus* MetRS (GkMetRS), we cloned the respective *metS* genes and evaluated the methionylation activity toward cognate tRNA^Met^ and near-cognate tRNA^Ile2^ substrates. We found that BcMetRS can efficiently discriminate between these two tRNAs despite the shared CAU anticodon, with an approximate 600-fold preference for tRNA^Met^ (Fig. 4). While not as efficient as BcMetRS, PaMetRS still displayed a moderate 80-fold discrimination, as did GkMetRS with 50-fold discrimination. That bacterial MetRS orthologs discriminate to any degree against near-cognate tRNA^Ile2^ suggests that nucleotides outside the anticodon act as negative determinants for methionylation.

The ability for each cloned TilS enzyme to recognize its near-cognate tRNA^Met^ substrate was also tested for all soluble proteins. While MetRS orthologs exhibit a range of tRNA_CAU_ discrimination (19), the TilS enzymes were largely unable to use tRNA^Met^. The EcTilS enzyme was able to use the tRNA^Met^ as a substrate for lysidinylation only under high protein and/or high tRNA concentrations with a >300-fold discrimination, as previously demonstrated (20).

## Discussion

Our understanding of the forces driving translational precision *versus* noisy gene expression remains murky. While it had been long assumed that mistranslation must be minimal to prevent a fatal “error catastrophe,” in many cases, it is now understood that cells can accommodate varying levels of imprecision, and indeed loosened stringency can be advantageous when environmental conditions fluctuate (28). An alternative hypothesis is that controls for translational fidelity can directly or indirectly influence transcript abundance or durability in ways that help cells adapt to variable environments (29).

Post-transcriptional modifications control numerous aspects of the tRNA life cycle, including ribosomal turnover rate, decoding preference, and AARS interactions. Some tRNA modifications serve such a significant role that they, and the genes encoding their biosynthetic enzymes, can be essential to the cell. Here, we probe features of TilS, the bacterial-specific enzyme responsible for synthesis of lysidine, by comparing BcTilS to several orthologous enzymes from varying genetic backgrounds.

The fidelity of tRNA-modifying enzymes is ensured through the use of identity elements in the tRNA substrate (3). We show that EcTilS is fully capable of modifying BctRNA^Ile2^, which shares all the previously determined EctRNA^Ile2^ identity elements (20). Yet BcTilS is unable to use EctRNA^Ile2^ as a substrate. We used a gain-of-function mutation strategy where elements of BctRNA^Ile2^ were sequentially transplanted into an EctRNA^Ile2^ scaffold (Fig. 1). Two constructs each recovered some activity: a base pair transversion in the acceptor stem (C3:G70 → G3:C70) and replacement of the D-loop. When combined, these two elements together produced a chimeric tRNA that could be lysidinylated as efficiently by BcTilS as the wildtype BctRNA^Ile2^. The 3:70 position is not an identity element for EcTilS, consistent with the observation that EcTilS rejects EctRNA^Met^ despite sharing the C3:G70 base pair with EctRNA^Ile2^. In contrast, BctRNA^Ile2^ and BctRNA^Met^ have different base pairs at the 3:70 position (G3:C70 and C3:G70, respectively); this difference helps prevent lysidinylation of a near-cognate tRNA. Transplanting the 11-nt *B*. *cenocepacia* D-loop onto EctRNA^Ile2^ (replacing an 8-nt D-loop) only modestly recovered activity but may help position the 3:70 base pair for BcTilS recognition, as the two substitutions together accounted for more than the sum of their respective activity gains.

The 10 bacterial genomes we evaluated here exhibit a wide range of AUA codon usage. We considered that AUA codon usage might be a predictor of TilS catalytic rate, such that organisms with more AUA codons would require more robust enzyme activity. While some bacteria with low AUA codon usage had lower catalytic efficiency, we identified no definitive correlation between codon usage and TilS efficiency (Table 1). We then postulated that our identification of AUA genes may have been too broad and shifted our analysis to AUA codon usage in HEGs (24). While AUA remains a minor codon in HEGs, the range of use is even more disparate than for genome-wide codon sequences, including some species that lack AUA in their HEGs entirely. The lack of correlation between catalytic efficiency and codon usage is exemplified by the BfTilS ortholog, which has higher AUA usage than *E*. *coli* in both HEGs and total coding sequences but lower activity.

It is unclear whether EcTilS is exceptionally efficient or BfTilS is an underperforming enzyme. One challenge to comparing TilS activity across species is that our *in vitro* comparisons use unmodified transcripts, and the constellation of other modifications is not known for mature tRNA^Ile2^ species. While Ikeuchi *et al*. (20) showed a relatively insignificant difference between fully modified (cellular) tRNA^Ile2^ and unmodified transcripts as EcTilS substrates, it could be that some orthologs require additional modifications before tRNA^Ile2^ can be recognized and lysidinylated. One candidate could be t^6^A_37_ just outside the anticodon, which enhances EcTilS activity (30). The interplay between multiple base modifications within a single tRNA species is likely to depend on growth rate and intracellular tRNA abundance, among other factors, which may influence the need for a more efficient TilS enzyme. In addition, transcriptional or translational upregulation may compensate for an intrinsically inefficient enzyme when cellular conditions demand, or activity may be increased by a yet-unidentified effector protein.

Given that tRNA^Ile2^ is initially transcribed with a CAU anticodon, the most direct challenge to translational accuracy is presumed to be misidentification of this tRNA as a methionine acceptor. We previously demonstrated that despite the CAU anticodon being an established identity element for MetRS, the ability of bacterial MetRS homologs to use unmodified tRNA^Ile2^ was variable, with discrimination against tRNA^Ile2^ ranging from 2- to 2000-fold (19). We have now added three more MetRS homologs to this list, and all fall within the previously determined range (Fig. 4). We considered that TilS enzymes might act on tRNA^Met^ with similar variability. However, despite having a CAU anticodon, the near-cognate tRNA^Met^ could not be lysidinylated to a measurable degree by any TilS we tested except for EcTilS, as previously shown (20). This low level of activity corresponds to >300-fold discrimination against the near-cognate tRNA^Met^, although *E*. *coli* MetRS displays only a 60-fold preference for tRNA^Met^ over tRNA^Ile2^. It is not clear whether a low level of tRNA^Met^ lysidinylation by EcTilS *in vitro* has cellular significance.

While over 98% of bacteria contain the *tilS* gene, suggesting its metabolic essentiality, we previously identified four evolved mutations outside the catalytic domain of BcTilS that each enhanced cellular fitness while decreasing enzyme function *in vitro* and *in vivo* (22). The conservation at these four positions ranges from 49% (position 1) to 77% (position 3) across 150 species evaluated (22). In the comparative study here, we recapitulated those mutations by making site-specific substitutions corresponding to the evolved mutations (Table S1) or by replacing the encoded amino acid with alanine (or with serine when the encoded amino acid was alanine; Table S2). Some variants could not be evaluated, as substitutions resulted in insoluble (PaTilS) or low-purity (HpTilS) proteins. In general, the evolved BcTilS mutations were functionally impaired to a greater degree than corresponding variants in orthologous proteins. This is not surprising, as *B*. *cenocepacia* was the organism subjected to selection; each individual mutation produced a fitness gain by reaching log-phase growth earlier alongside reduced TilS enzyme activity. Of the orthologs studied here, GkTilS was the most resistant to substitutions, with no more than a twofold loss in activity at any position tested (Fig. 3). The robustness of GkTilS activity may be related to the nature of *Geobacillus kaustophilus* as a thermophilic bacterium that tolerates wide ranges of pH and salinity in addition to temperature (31). It may be that GkTilS is more structurally tolerant, as likely required in its extreme native environment.

Alanine substitutions had minimal impact on catalytic activity across the homologs, except EcTilS-L227A and BfTilS-I232A (position #1), which had reduced activity, and MsTilS-R260A (position #2), which had no detectable activity. Interestingly, the other position 1 residues were alanine in each wildtype TilS homolog, and even substitution to serine reduced activity less than twofold for each variant, suggesting that wildtype side chains at these positions do not contribute to specific networks within the protein structure.

Position #3 contains a proline in each of the homologs studied here except for BfTilS, where it is a serine. While the EcTilS P387L substitution did not lead to a loss in activity, we noted that position 388 is also a proline, suggesting possible redundancy in this protein. Some of the variants likely impact protein structure, as suggested by insoluble proteins EcTilS T412K, BfTilS D412K, and BfTilS D412A, all position #4 substitutions.

The evolved BcTilS mutations leading to increased fitness despite decreased activity are not part of the N-terminal catalytic domain (Fig. 3). While only GkTilS has been crystalized in complex with tRNA^Ile2^, overall sequence conservation suggests structural similarities with TilS orthologs. In contrast with anticodon-modifying enzymes that only read out anticodon nucleotides (*e*.*g*., MnmA (32), QueTGT (33), and TadA (34)), TilS uses one or more C-terminal appended domains (type II or type I enzymes, respectively) to contact the anticodon and/or acceptor stems of its cognate tRNA. Other tRNA-acting enzymes like the AARSs use both direct and water-mediated hydrogen bonding with atomic groups in the tRNA or shape complementarity with the tRNA structure (35, 36). We have recently demonstrated that BcTilS and EcTilS (both type I representatives) use nucleotides in the tRNA acceptor stem to differentiate between CAU-containing tRNA^Ile2^ and tRNA^Met^ (21). Evolved mutations at positions 1 and 2 are near the anticodon stem, and positions 3 and 4 are near the acceptor stem in the GkTilS–tRNA^Ile2^ cocrystal structure, but none of the residues directly contacts the tRNA. Position 3, the evolved mutation resulting in BcTilS P421L, is conserved as a proline in ∼77% of bacterial sequences analyzed (22), yet substitution to P421A is only minimally detrimental to enzyme activity. The driving force for loss of activity because of this mutation is therefore not as simple as a rigid residue being replaced by one with greater conformational flexibility. We expect that side-chain substitutions at these distal positions may influence the structural ensemble and allow catalytically active conformations to be more frequently sampled (37). Comparison of the GkTilS–tRNA^Ile2^ cocrystal structure with the EcTilS apoenzyme structure clearly indicates a conformational rearrangement of the CTDs relative to the N-terminal active site upon tRNA binding, with the long α-helical linker serving as a hinge (23).

Given the conservation of TilS in bacteria and its single known cellular target, we were surprised at the range of activities observed here and apparent lack of correlation with genomic characteristics. Measured rates do not precisely align with genomic G-C content or TilS type I/II classification, and amino acid substitutions in presumed structurally similar regions have widely different effects on enzyme activity. Maybe most surprising is a lack of direct correlation with genome-wide or HEG AUA frequency. We anticipated that AUA frequency would be a major contributor to TilS activity, but that was not borne out in these *in vitro* experiments, except for the observation that the species lacking AUA in HEGs did have the least efficient TilS activity. The questions that arose offer new possibilities to explore. As lysine is a substrate for TilS, it may be that some organisms use TilS to sense amino acid availability through lysine abundance. This would be consistent with the significance of lysine availability in our prior observation that the fitness deficit of wildtype *B*. *cenocepacia* relative to evolved mutants is relieved by lysine supplementation (22).

It is interesting to note that while class I TilS proteins are typically >400 amino acid long (and class II are >300 amino acids), the low AUA-using, low-efficiency homologs have only one (PaTilS and BcTilS) or two (MsTilS) lysines in their sequences. In contrast, the high AUA-using BbTilS and MpTilS each have almost 50 lysine residues; unfortunately, we were not able to obtain kinetic data for the latter two homologs. It is possible that TilS plays an uncharacterized role in regulation, which could correlate with GC content, as high GC genomes are under-represented in AUA codons and might depend less on robust TilS activity. The links between TilS activity, amino acid starvation, and gene regulation are questions of interest currently being explored.

We also determined here that the enzymes tested (EcTilS and BcTilS) do not use the same tRNA nucleotides as identity elements. As bacterial genome evolution solved the AUN codon box challenge, introduction of tRNA^Ile2^ with its CAU anticodon brought a new dilemma of discriminating against tRNA^Met^. While MetRS exhibits a range of selectivity for tRNA^Met^ *versus* tRNA^Ile2^, TilS seems more selective for its cognate tRNA, at least *in vitro*.

TilS is not solely responsible for accurate AUA decoding, however. A wobble-modifying enzyme was recently identified that targets the C34 position of elongator tRNA^Met^ in many bacteria and some archaea (38). tRNA^Met^ cytidine acetate ligase (TmcAL) activates an acetate ion with ATP to generate an acetyladenylate and subsequently installs the acetyl group at the wobble base N4 position. Just as L34 in the modified tRNA^Ile2^ prevents decoding of the AUG codon, so the presence of ac^4^C34 tRNA^Met^ introduces a steric block with the AUA codon. Suzuki *et al*. demonstrated that TmcAL and TilS cooperate to ensure faithful translation of the AUA codon with isoleucine; when both C34-modifying enzymes are lacking, incorporation of methionine at AUA codons increases and a cold-sensitive phenotype appears. While TilS uses identity elements in the tRNA^Ile^ acceptor stem, TmcAL discriminates using anticodon loop nucleotides 32 and 38 (38). We presume that MetRS evolved earlier than either TilS or TmcAL, as TilS exists only in bacteria and TmcAL is present even more rarely across species. It seems likely that tRNA^Met^ identity elements were fixed before the appearance of TilS and TmcAL, which together partition CAU-containing tRNAs into isoleucine and methionine acceptors.

The puzzle as to what drove fitness-enhancing mutations in BcTilS remains. BcTilS has no AUA codons in its HEGs and very few in other genes; nevertheless, there may be one or more key proteins yet to be identified whose expression using the lysidinylated tRNA^Ile2^ is a regulatory signal. Alternatively, cells may sacrifice translational accuracy for adaptation during nutritional or other stress conditions. We are currently investigating whether misincorporation of methionine arising from BcTilS mutants may be protective under oxidative conditions, as has been shown in other systems (39, 40, 41, 42). The range of TilS activities described here may reflect the varying environments occupied by the bacterial species sampled more than AUA usage, and it remains possible that the *tilS* gene is not essential in all species.

## Experimental procedures

### Purification of recombinant TilS proteins

The *tilS* genes from select bacteria were cloned from genomic material into pET-28a (Invitrogen) for expression in Rosetta II *E*. *coli* cells. A detailed explanation of the cloning and purification, including sources of bacterial strains or genomic material and alternative cloning strategies used, can be found in Supporting Information.

### Preparation and radiolabeling of transcript tRNA

The tRNA^Ile2^ transcripts were synthesized as previously described by Sherlin *et al*. (43) using sequences obtained from the Genomic tRNA database (44). Double-stranded tDNA was constructed using the large Klenow fragment of DNA polymerase I (NEB) from overlapping oligonucleotides (IDT DNA) carrying a T7 RNA polymerase promoter upstream of the tRNA template. T7 RNA polymerase transcription of the tDNA was allowed to proceed overnight at 37 °C in 200 mM Tris–HCl (pH 7.5), 30 mM MgCl_2_, 0.1 mg/ml bovine serum albumin, 2 mM spermidine, 40 mM DTT, and 5 mM each NTP. Following overnight transcription, tDNA templates were removed by RNase-free DNase (Thermo Scientific) digestion for 1 h at 37 °C. The remaining nucleic acid was concentrated by ethanol precipitation and resuspended in 10 mM Tris–HCl (pH 7.5) and 1 mM EDTA (TE buffer). Transcription products were separated on a 7 M urea/10% polyacrylamide gel, and tRNAs were eluted from crushed excised gel fragments in 500 mM ammonium acetate (pH 5.3), 1 mM EDTA. Following gel elution, samples were ethanol precipitated and resuspended in TE buffer. tRNA transcripts for electrophoretic mobility shift assays were ^32^P-radiolabeled at the 3′-internucleotide linkage with [α-^32^P] ATP using an ATP–PP_*i*_ exchange with *E*. *coli* tRNA nucleotidyltransferase as previously described (45, 46). Final ^32^P-labeled tRNA products were stored in TE buffer at −20 °C.

### Lysidinylation of transcript tRNA

tRNA samples were annealed by heating to 80 °C for 5 min followed by the addition of 10 mM MgCl_2_ and cooling to ambient temperature. Before initiating steady-state kinetics, we determined the pH optimum for each TilS. Each enzyme exhibited maximum activity at pH 8.5 or 9.0; these findings are consistent with the study by Salowe *et al*. (47). Lysidinylation of tRNA^Ile2^ was carried out in 100 mM Tris–HCl (pH 8.5 or 9.0), 5 mM DTT, 10 mM MgCl_2_, 10 mM KCl, 2 mM ATP, 9.75 μM ^3^H-lysine (Moravek Biochemicals, Inc), tRNA (0.2–20 μM BctRNA^Ile2^, 0.2–20 μM EctRNA^Ile2^, 1.0–50 μM BftRNA^Ile2^, 0.4–20 μM MstRNA^Ile2^, 0.5–18 μM HptRNA^Ile2^, 1.0–25 μM PatRNA^Ile2^, and 0.05–10 μM GktRNA^Ile2^), and 5 μM L-lysine. EcTilS (50 nM) was assayed using aliquots removed at 15-s intervals for 1 to 3 min. BfTilS (100 nM) was assayed using aliquots removed at 15-s intervals for 1.5 min. MsTilS (500 nM) was assayed using aliquots removed at 2.5 min and then 5-min intervals for 15 min. HpTilS (100 nM) was assayed using aliquots removed at 15-s intervals for 1 min. PaTilS (100 nM) was assayed using aliquots removed at 2.5-min intervals for 15 min. GkTilS (500 nM) was assayed using aliquots removed at 15-s intervals for 1.5 min. The less active BcTilS (500 nM) was assayed with samples collected at 15 min intervals for 90 to 180 min. Each reaction aliquot was quenched by spotting on Whatman filter paper previously soaked in 5% trichloroacetic acid and dried. Filters were counted by liquid scintillation to quantify lysine incorporation into transcript tRNA. Steady-state kinetic parameters were acquired by fitting initial velocities for product (tRNA^Ile2^_LAU_) formation to a Michaelis–Menten curve using GraphPad Prism software (version 9.4; GraphPad Software, Inc). Relative rates were determined by comparing linear initial velocities of TilS or tRNA variants with the wildtype reaction.

### Electrophoretic mobility shift assay

TilS proteins were incubated with 3.5 nM ^32^P-labeled tRNA^Ile2^ at 37 °C for 30 min in 100 mM Tris–HCl (pH 7.8), 5 mM DTT, 10 mM MgCl_2_, 10 mM KCl, and 10% glycerol (10 μl reaction volume). Protein ranges used were as follows: BcTilS (0.2–30 μM), EcTilS (0.2–30 μM), BfTilS (0.4–52 μM), MsTilS (0.6–75 μM), PaTilS (0.4–52 μM), HpTilS (0.07–8.5 μM), GkTilS (0.07–9.5 μM). The TilS–tRNA complex was separated from free tRNA on a native 10% polyacrylamide gel at 165 V for 20 min. Gels were blot dried and exposed overnight to a phosphorimager screen. Screens were analyzed using an Amersham Biosciences Typhoon IP phosphorimager and ImageQuant v8.2 software. The percent of tRNA complexed with TilS was plotted against TilS protein concentration. Data were fit to a sigmoidal binding curve using the Hill equation in GraphPad Prism (version 9.4) software to identify *B*_Max_ and *K*_*d*_ for each protein.

### Comparison of genomic features associated with AUA decoding

Prokaryotic genome sequences were obtained from the National Center for Biotechnology Information genome database (48). Codon usage and G:C content in ORFs of *B*. *cenocepacia* (HI2424), *E*. *coli* (W3110), *M*. *smegmatis* (MG(2)155), *P*. *aeruginosa* (PAO1), *B*. *fragilis* (NCTC9343), *H*. *pylori* (26695), and *G*. *kaustaphilus* (HTA426) genomes were obtained from the Kazusa codon usage database (49). GenBank assembly sequences were uploaded to the Dynamic Codon Biaser web server to determine codon usage statistics in HEGs in each of the seven prokaryotic genomes (50). The number of tRNA^Ile2^ gene copies was identified from the Genomic tRNA Database (44).

## Data availability

Additional data can be found in the supporting information (23, 51, 52, 53, 54, 55, 56, 57).

## Supporting information

This article contains supporting information (23, 51, 52, 53, 54, 55, 56, 57).

## Conflict of interest

The authors declare that they have no conflict of interest with the contents of this article.
