# Supplementary material for: The bacterial tRNA-modifying enzyme tRNAIle lysidine synthetase is genetically conserved but catalytically variable
Source: J Biol Chem. 2025 Sep 4;301(10):110688. doi: 10.1016/j.jbc.2025.110688 (PMC12514573; doi:10.1016/j.jbc.2025.110688)
Supplement: Supplementary Materials [file mmc1.docx]

**The bacterial tRNA modifying enzyme tRNA^Ile2^ lysidine synthetase is genetically conserved but catalytically variable**

Marc J. Muraski^1^, Ferdiemar C. Guinto, Jr.^1^, Emil M. Nilsson^1,2^, Jorge B. Dabdoub^1^, Samantha C. Robinson^1,3^, Yiyang Jiang^1^, Zhen Shen^1^, Rebecca W. Alexander^1*^

^1^Department of Chemistry and Center for Molecular Signaling, Wake Forest University, Winston-Salem, NC 27109

^2^Pharmaceutical Services Group, ThermoFisher, High Point, NC 27265

^3^Department of Chemistry, University of California Davis, Davis, CA 95616

* Correspondence: [alexanr@wfu.edu](mailto:alexanr@wfu.edu)

**Supporting Information**

**Materials and Methods**

Purification of recombinant TilS proteins

The *tilS* genes from select bacteria were cloned from genomic material into pET-28a (Invitrogen) for expression in Rosetta II *E. coli* cells. Bacterial strains used were *Burkholderia cenocepacia* strain HI2424 (gift from V. Cooper, University of Pittsburgh School of Medicine), *Escherichia coli* strain K-12, *Mycobacterium smegmatis* strain MC(2)155 (gift from P. Dos Santos, Wake Forest University), *Bacteroides fragilis* strain NCTC9343 (ATCC)*, Borrelia burgdorferi* strain B31 (ATCC)*, Mycoplasma penetrans* strain HF-2 (gift from M. Balish, Miami U. of Ohio), *Helicobacter pylori* strain 26695 (gift from T. Hendrickson, Wayne State University), *Pseudomonas aeruginosa* (gift from M. Lee-Brown, Guilford College). Genomic DNA of *Geobacillus kaustophilus* JCM 20319T was provided by the RIKEN BRC through the National BioResource Project of the MEXT. The *tilS* genes from *Staphylococcus* strain R6, *M. penetrans* strain HF-2, and *G. kaustophilus* strain HTA426 were synthesized and cloned into pET-28a through the Genewiz cloning service with codon optimization. The *tilS* gene from pET-28a:HpTilS was subcloned into pBAD (Invitrogen) in house, while the *tilS* genes from pET-28a:MpTilS, pET-28a:SpTilS, and pET-28a:BbTilS were subcloned into pBAD through the Genewiz cloning service. A list of the primers used in this work for cloning and mutagenesis is provided (Table S5). Plasmids were transformed into chemically competent Rosetta II cells for pET-28a or Top10 for pBAD. Cells containing a pET-28a construct were grown at 37 ⁰C to an OD_600_ of 0.6; protein expression was induced by the addition of Isopropyl-β-D-thiogalactopyranoside (IPTG, Sigma) to 0.1 mM or 1 mM and harvested two to four hours post induction. Cells containing the pBAD:HpTilS construct were grown at 37 ⁰C to OD_600_ of 0.8, the temperature was reduced to 25 ⁰C and protein overexpression was induced by the addition of L-arabinose (ThermoFisher) to 0.2 % and harvested 16 hours post induction. Proteins were purified by Ni-affinity chromatography on His-trap columns (GE) following the manufacturer's protocol. The pBAD:HpTilS purification required a wash with a chaperone stripping buffer prior to elution (20 mM Tris, pH 8.0; 100 mM KCl, 300 mM NaCl, 5 mM ATP, 20 mM MgCl_2_,10% glycerol) for 10 column volumes at a flow rate of 4 mL/min (1). Proteins were purified to greater than 95% homogeneity, as determined by SDS-PAGE analysis, and stored at -20 °C in 40 mM Tris-HCl (pH 8.5), 200 mM NaCl, 20 mM MgCl_2_, 20 mM KCl, and 40% glycerol for BcTilS and all others in 50 mM Tris-HCl (pH 8.5), 400 mM NaCl, 5 mM β-mercaptoethanol, and 40% glycerol.

Size exclusion chromatography multi-angle light scattering

The approximate molecular weights of BcTilS, EcTilS, and BfTilS were determined using size exclusion chromatography coupled with multi-angle light scattering (SEC-MALS) (2, 3). Recombinant TilS (1 mg/mL) was equilibrated in SEC-MALS buffer (50 mM sodium phosphate·NaOH (pH 8.0), 250 mM NaCl, 5 mM β-mercaptoethanol, 5% glycerol). The protein was resolved using an analytical size exclusion TSK gel column (7.8 mM x 30 cm, 8 mM particle size; Tosoh Bioscience) at a flow rate of 0.5 mL/min over 35 minutes. Elution was monitored by UV_280_ absorbance and molecular mass determined by MALS (HELIOS II; Wyatt Technology) with linked refractive index determined (Oprilab rEX; Wyatt Technology). Astra 6.1 software (Wyatt Technology) was used to analyze peaks based on UV_280,_ and figures were generated using GraphPad Prism (v9.4).

**Results**

Cloning and purification of TilS orthologs

A library of *tilS* genes (listed above) was cloned into the pET-28a construct; site-directed mutagenesis was used to investigate the catalytic importance of residues within the TilS enzyme. The BcTilS construct and its mutants were robust in their overexpression except for N274Y, which required a 10-fold reduction in IPTG to enable expression of soluble protein. The EcTilS, MsTilS, and BfTilS constructs all expressed well other than EcTilS-T412K and BfTilS-D408K(A), this position seemed to be difficult to express and often resulted in insoluble proteins following cellular lysis. Additionally, BfTilS displayed a loss of activity during long-term storage, requiring enzymes to be purified and used within the week. The PaTilS wild-type construct purified with a chaperone protein unless expressed with 0.1 mM IPTG for only two hours rather than four; most PaTilS variants were insoluble regardless of IPTG concentration. Similar to PaTilS, the GkTilS (non-synthetic) construct and its mutants required the lower IPTG concentration and 2-hour expression time; this enabled expression of wild-type enzyme and variants for this ortholog. In general, most mutant constructs did not produce as much soluble protein as the wild-type *tilS.*

The remaining constructs (HpTilS, MpTilS, BbTilS, and SpTilS) all resulted in insoluble proteins when expressed in pET-28a regardless of IPTG concentration, temperature, or expression time. We considered that placement of the histidine tag close to the active site could be impairing expression; however, C-terminal tag constructs were also insoluble. We tried subcloning these *tilS* genes into the pBAD construct, as it has more tunability with the arabinose inducer and uses the host polymerase rather than the dedicated T7 polymerase. Although the HpTilS construct generated soluble protein using this strategy, it co-purified with the GroEL chaperone. GroEL was removed post isolation on a nickel IMAC column. Based on this result, no mutagenesis was attempted for this ortholog. Expression and purification for BbTilS and SpTilS were variable under these conditions and generally contained numerous impurities; further, the tRNA transcripts for these species did not transcribe well with suitable purity. Based on the findings of Konczal *et al* we considered the codon optimization performed during synthesis of MpTilS, GkTilS, and SpTilS could be impairing expression (4). The *G. kaustophilus* *tilS* gene was later lifted from genomic material and cloned into pET-28a. While it still required lower IPTG and shorter expression time, the overall expression was improved by this change for several of the mutations. The *M. penetrans* *tilS* was also lifted from genomic material and cloned into pET-28a; it required five UGA codons to be converted to UGG by PCR mutagenesis, as the mycoplasma genus utilizes this codon as a tryptophan, while in *E. coli* this is the amber stop codon. Unlike for GkTilS, the use of a non-optimized gene did not improve the expression for this gene.

Oligomeric state of TilS orthologs

We predicted that the cloned TilS orthologs would be dimeric, as the available crystal structures from *Geobacillus kaustophilus* (3A2K)*, Aquifex aeolicus* (2E89)*,* and *Escherichia coli* (1NI5) are all dimeric (5, 6). Of these proteins, GkTilS and EcTilS are examples of Type I TilSs, with an extended C-terminal domain known as CTD2. In contrast, AaTilS is a Type II TilS, which has a shorter C-terminal domain. We investigated the oligomeric state of TilS orthologs using SEC-MALS to assess their apparent molecular weight. The monomeric molecular weights were calculated using Expasy ProtParam, including the encoded hexahistidine tag and linker (7). These values were compared to the molecular weight identified using the Mp value from the ASTRA 6 analysis software. The BcTilS protein produced two peaks; the major peak had a molecular weight of 87.3 kDa and a minor peak of 130.2 kDa corresponding to 1.6-fold and 2.4-fold higher than the predicted monomeric weight, respectively **(Figure S2)**. The EcTilS protein exhibited an apparent molecular weight of 86.8 kDa, while BfTilS was 115.3 kDa; these are 1.7-fold and 2.2-fold higher than the predicted monomeric weights, respectively. From these values the molecular weights for the TilS orthologs tested were between 1.6 and 2.2-fold higher than the predicted monomeric weights, consistent with a dimeric organization for these proteins. The peak at approximately 25 minutes corresponds to the BME in the samples.





**Figure S1. Electrophoretic mobility shift assay.**

Representative gels from each EMSA are shown utilizing radiolabeled ^32^P-tRNA^Ile2^ [3.5 nM]. The right-most well lacks protein as a control and left to right is increasing concentrations of TilS enzyme. A) BcTilS [0.2-30 μM]; B) EcTilS [0.2-30 μM]; C) BfTilS [0.4-52 μM]; D) HpTilS [0.07-8.5 μM]; E) MsTilS [0.6-75 μM]; F) PaTilS [0.4-52 μM]; G) GkTilS [0.07-9.5 μM].


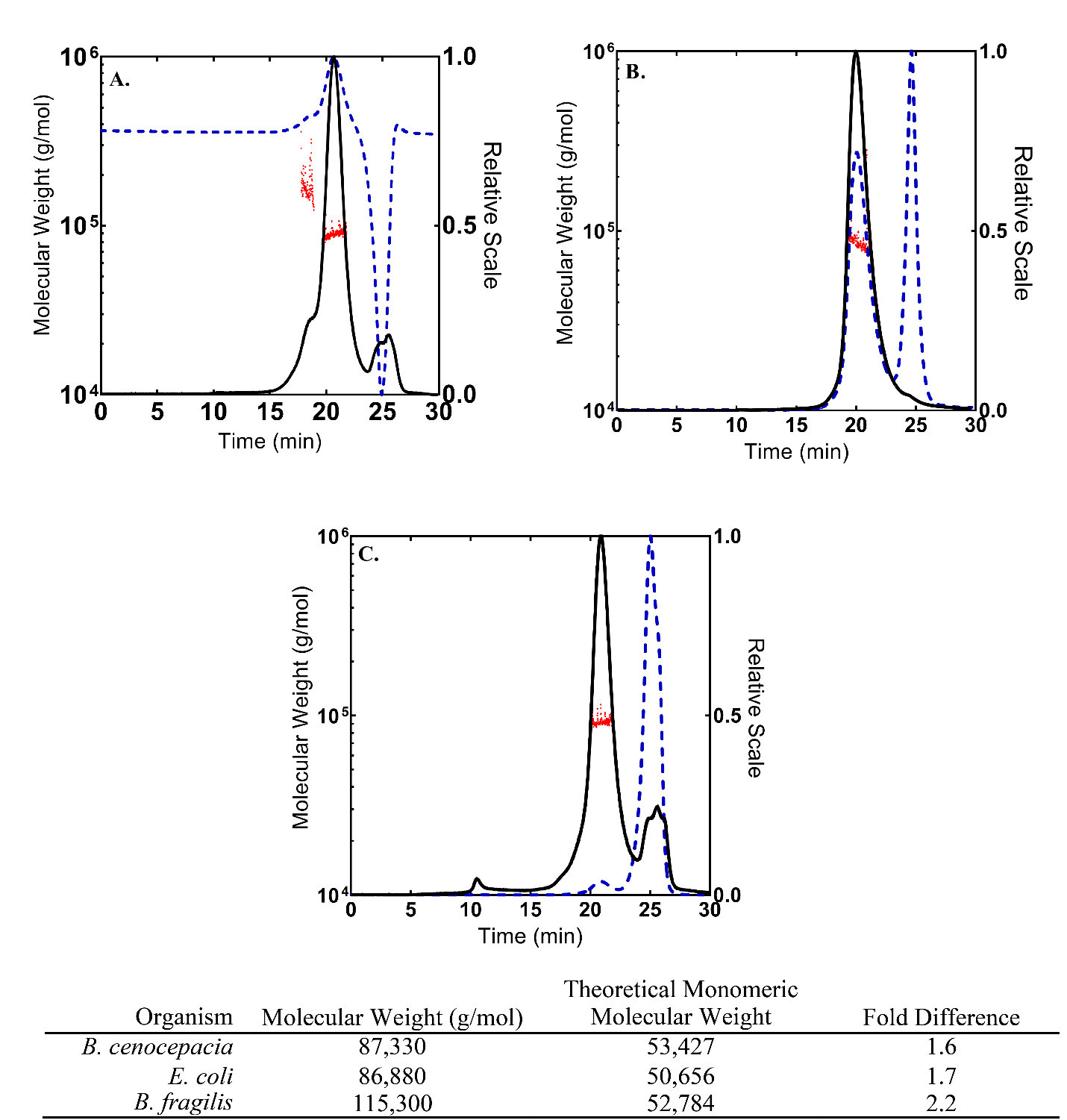


**Figure S2. Determination of TilS oligomeric state.**

BcTilS, EcTilS, and BfTilS (each 1 mg/mL) were separated by HPLC and passed through a Wyatt Dawn Helios-II and Optilab rEX to measure light scattering and refractive index of the protein. The absorbance at 280 nm was monitored by a Waters 2417 absorbance detector. SEC-MALS analysis identified molecular weights at peak reading of 87.3 kDa and monodisperse for BcTilS (panel A), 86.9 kDa and monodisperse for EcTilS (panel B), and 115.3 kDa and monodisperse for BfTilS (panel C). Solid line, A_280_ trace; dashed line, differential refractive index (dRI); and dots, molecular weights. Molecular weights are plotted on the left y-axis while the UV and dRI traces were normalized to 1 and plotted on the right y-axis.


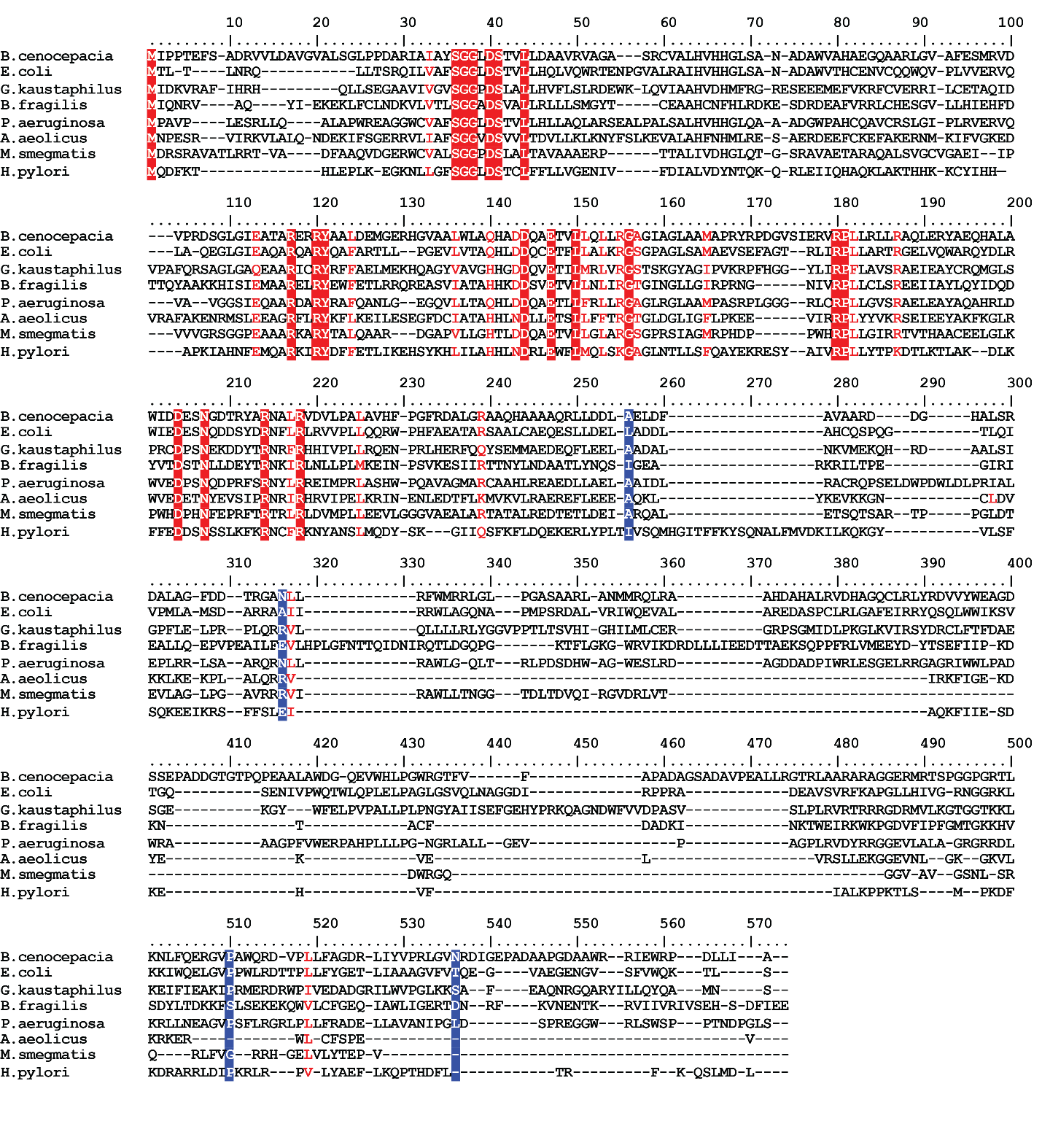


**Figure S3. Representative TilS sequences.**

Multiple sequence alignment of TilS homologs used in this work and *A. aeolicus*, a Type II TilS for which a crystal structure has been solved (PDB ID #2E21; (6)). Residues that are chemically similar are in red text, chemical identity are highlighted red with white text and sites that were used in site-directed mutagenesis are highlighted blue with white text. Multiple sequence alignment was generated using T-coffee MSA service (8).

**Table S1. Relative rate of evolved mutant residues**

| **BcTilS** |  | **WT** | **A244T** | **N274Y** | **P421L** | **N445K** |
| --- | --- | --- | --- | --- | --- | --- |
|  | Lysidinylation Rate (nM/min) | 5.8 ± 1.9 | 0.17 ± 0.02 | 0.08 ± 0.02 | 0.03 ± 0.02 | 0.8 ± 0.1 |
|  | Relative Rate | 100 | 3.4 ± 0.8 | 1.6 ± 0.2 | 0.6 ± 0.4 | 14.8 ± 5.8 |
|  | Catalytic Loss (Fold) | 1 | 34.1 | 72.8 | 167 | 7.0 |
| **EcTilS** |  | **WT** | **L227T** | **A256Y** | **P387L** | **T412K** |
|  | Lysidinylation Rate (nM/min) | 139.0 ± 9.8 | 26.5 ± 1.0 | 45.7 ± 0.6 | 171.9 ± 4.2 | ND |
|  | Relative Rate | 100 | 19.6 ± 1.2 | 33.8 ± 2.1 | 127 ± 7 | ND |
|  | Catalytic Loss (Fold) | 1 | 5.2 | 3.0 | 0.8 | ND |
| **GkTilS** |  | **WT** | **A239T** | **Q274Y** | **P416L** | **S442K** |
|  | Lysidinylation Rate (nM/min) | 92.5 ± 2.6 | 84.7 ± 26 | 120.0 ± 10.0 | 50.5 ± 9.9 | 56.9 ± 7.5 |
|  | Relative Rate | 100 | 92 ± 32 | 131 ± 12 | 55 ± 12 | 63 ± 7 |
|  | Catalytic Loss (Fold) | 1 | 1.1 | 0.8 | 1.8 | 1.6 |
| **BfTilS** |  | **WT** | **I232T** | **E262Y** | **S383L** | **D412K** |
|  | Lysidinylation Rate (nM/min) | 68.9 ± 18.2 | 10.3 ± 7.9 | 68.5 ± 9.9 | 1.3 ± 1.4 | ND |
|  | Relative Rate | 100 | 15 ± 12 | 90 ± 16 | 1.3 ± 1.4 | ND |
|  | Catalytic Loss (Fold) | 1 | 6.7 | 1 | 54.8 | ND |
| **MsTilS** |  | **WT** | **A228T** | **R260Y** |  |  |
|  | Lysidinylation Rate (nM/min) | 59.2 ± 25.1 | 12.1 ± 7.5 | 8.0 ± 3.4 | NA | NA |
|  | Relative Rate | 100 | 23 ± 14 | 15.5 ± 5.9 | NA | NA |
|  | Catalytic Loss (Fold) | 1 | 5.7 | 8.7 | NA | NA |

* Average of at least 3 individual trials ± standard deviation; ND (Insoluble); NM (Not Measurable); NA (Residue Missing)

**Table S2. Relative rate of alanine mutant residues**

| **BcTilS** |  | **WT** | **A244S** | **N274A** | **P421A** | **N445A** |
| --- | --- | --- | --- | --- | --- | --- |
|  | Lysidinylation Rate (nM/min) | 5.8 ± 1.9 | 3.7 ± 0.4 | 0.83 ± 0.06 | 3.3 ± 1.5 | 9.2 ± 1.4 |
|  | Relative Rate | 100 | 70 ± 17 | 16 ± 6 | 55 ± 26 | 176 ± 39 |
|  | Catalytic Loss (Fold) | 1 | 1.6 | 7 | 1.8 | 0.6 |
| **EcTilS** |  | **WT** | **L227A** | **A256S** | **P387A** | **T412A** |
|  | Lysidinylation Rate (nM/min) | 139 ± 4.9 | 5.2 ± 0.3 | 137.3 ± 10.0 | 172.2 ± 28.0 | 185 ± 20 |
|  | Relative Rate | 100 | 3.8 ± 0.2 | 99 ± 12 | 126 ± 23 | 130 ± 7 |
|  | Catalytic Loss (Fold) | 1 | 26.8 | 1 | 0.8 | 0.8 |
| **GkTilS** |  | **WT** | **A239S** | **Q274A** | **P416A** | **S442A** |
|  | Lysidinylation Rate (nM/min) | 92.5 ± 1.3 | 61.3 ± 8.5 | 100.5 ± 6.8 | 66 ± 14 | 46 ± 13 |
|  | Relative Rate | 100 | 67 ± 11 | 109 ± 7 | 72 ± 17 | 50 ± 14 |
|  | Catalytic Loss (Fold) | 1 | 1.5 | 0.9 | 1.4 | 2 |
| **BfTilS** |  | **WT** | **I232A** | **E262A** | **S383A** | **D412A** |
|  | Lysidinylation Rate (nM/min) | 69 ± 18 | 7.4 ± 2.8 | 150 ± 53 | 22.6 ± 8.5 | ND |
|  | Relative Rate | 100 | 11 ± 4 | 217 ± 37 | 32 ± 15 | ND |
|  | Catalytic Loss (Fold) | 1 | 9.3 | 0.5 | 3.1 | ND |
| **MsTilS** |  | **WT** | **A228S** | **R260A** |  |  |
|  | Lysidinylation Rate (nM/min) | 59 ± 26 | 38 ± 3 | 0.4 ± 0.1 | NA | NA |
|  | Relative Rate | 100 | 62 ± 10 | 0.6 ± 0.3 | NA | NA |
|  | Catalytic Loss (Fold) | 1 | 1.8 | 189 | NA | NA |

* Average of at least 3 individual trials ± standard deviation; ND (Insoluble); NM (Not Measurable); NA (Residue Missing)

**Table S3. Residue conservation in TilS orthologs**

|  | Position #1 | | Position #2 | | | Position #3 | | | Position #4 | | |
| --- | --- | --- | --- | --- | --- | --- | --- | --- | --- | --- | --- |
| Organism | Residue | Conservation | | Residue | Conservation | | Residue | Conservation | | Residue | Conservation |
| *B. cenocepacia* | Ala244 | 49 % | | Asn274 | 10% | | Pro421 | 77% | | Asn445 | 3% |
| *E. coli* | Leu227 | 13% | | Ala256 | 9% | | Pro387 | 77% | | Thr412 | 3% |
| *G. kaustophilus* | Ala239 | 49 % | | Arg271 | 53% | | Pro416 | 77% | | Ser442 | 58% |
| *B. fragilis* | Ile232 | 4% | | Glu262 | 6% | | Ser383 | 6% | | Asp408 | 8% |
| *P. aeruginosa* | Ala234 | 49 % | | Asn273 | 10% | | Pro395 | 77% | | Leu420 | 0% |
| *M. smegmatis* | Ala228 | 49 % | | Arg260 | 53% | | Gly309 | 1% | | -- | -- |
| *H. pylori* | Ile219 | 4% | | Glu266 | 6% | | Pro306 | 77% | | -- | -- |
| *A. aeolicus* | Ala241 | 49 % | | Arg269 | 53% | | -- | -- | | -- | -- |
| **Consensus** | **Ala** | **49 %** | | **Arg** | **53%** | | **Pro** | **77%** | | **Ser** | **58%** |

**Table S4. Codon usage in highly expressed genes.**

|  | | | | AUA codons | | | | Ile codons | | |  |
| --- | --- | --- | --- | --- | --- | --- | --- | --- | --- | --- | --- |
|  | | **Organism** | **GC%** | **HEG codons analyzed** | HEG AUA codons | AUA/1000 HEG codons | AUA /1000 codons | % of genomic AUA usage | Ile/1000 HEG codons | Ile/1000 codons | % of genomic Ile usage |
| High | | *B. burgdorferi* | 28.8 | 6741 | 201 | 29.8 | 39.0 | 76.5 | 85.3 | 102.5 | 83.2 |
|  |  | *M. penetrans* | 26.5 | 4859 | 34 | 7.0 | 20.9 | 33.5 | 75.7 | 89.5 | 84.6 |
|  |  | *B. fragilis* | 44.1 | 6685 | 27 | 4.0 | 16.9 | 23.9 | 66.6 | 69.8 | 95.3 |
| Mid | | *H. pylori* | 39.6 | 6763 | 15 | 2.2 | 9.0 | 24.7 | 66.7 | 71.5 | 93.2 |
|  |  | *S. pyogenes* | 39.0 | 6816 | 3 | 0.4 | 7.2 | 6.1 | 62.9 | 73.6 | 85.5 |
|  |  | *E. coli* | 51.5 | 6794 | 1 | 0.2 | 4.9 | 3.1 | 68.8 | 59.1 | 116.5 |
|  |  | *G. kaustophilus* | 52.8 | 6727 | 3 | 0.5 | 2.9 | 15.5 | 68.8 | 63.5 | 108.4 |
| Low | | *P. aeruginosa* | 67.1 | 6799 | 0 | 0 | 0.9 | 0 | 52.8 | 41.6 | 126.9 |
|  |  | *M. smegmatis* | 67.8 | 7146 | 0 | 0 | 0.9 | 0 | 51.9 | 43.2 | 120.1 |
|  |  | *B. cenocepacia* | 67.4 | 6927 | 0 | 0 | 0.6 | 0 | 44.9 | 34.4 | 130.6 |

**Table S5. Primers used in cloning and mutagenesis**

Table S4. Cloning and mutagenesis primers used in this work.

*Borrelia burgdorferi*

| BbTilS lift primers |  |
| --- | --- |
| BbTilS lift forward | gacttgaaaaattgtatgattttttaagtgatgagctgattgatattagcg |
| BbTilS lift reverse | gctgaaattagaaaaacaacaacaagtcctaaaatcc |
|  |  |
| BbTilS cloning primers |  |
| BbTilS NheI forward | AAAAAAGCTAGCATGCATTTTTTAGACGAAAATATACAAATTAA |
| BbTilS XhoI reverse | AAAAAACTCGAGTCATTCCTTTAAAAGCCCTCCAATCAAAGATA |
|  |  |
| BbTilS His tag switch |  |
| NheI to NcoI forward | GCGGCAGCCATATGGCTACCATGGATTTTTTAGACGAAAATA |
| NheI to NcoI reverse | TATTTTCGTCTAAAAAATCCATGGTAGCCATATGGCTGCCGC |
| Remove stop forward | GATTGGAGGGCTTTTAAAGGAAGGACTCGAGCACCA |
| Remove stop reverse | TGGTGCTCGAGTCCTTCCTTTAAAAGCCCTCCAATC |
|  |  |
| tRNA primers |  |
| Bb tRNA^Ile2^ sequence | GGGCCCATAGCTCAGTTGGTTAGAGCACCCGACTCATAATCGGTAGGTCCCAGGTTCAAGTCCTGGTGGGCCCACCA |
| Bb tRNA^Ile2^ 5' | aattcctgcagTAATACGACTCACTATAGGGCCCATAGCTCAGTTGGTTAGAGCACCCGACTCATAATCGGT |
| Bb tRNA^Ile2^ 3' | TGGTGGGCCCACCAGGACTTGAACCTGGGACCTACCGATTATGAGTCGGG |
|  |  |
| Bb tRNA^Met^ sequence | GCTGATGTAGCTCAGTTGGTtAGAGCACTCGGCTCATATCCGAGTTGtCGTGGGTTCAAGTCCCTCCATCAGCAcca |
| Bb tRNA^Met^ 5' | aattcctgcagTAATACGACTCACTATAGCTGATGTAGCTCAGTTGGTTAGAGCACTCGGCTCATATCCGAG |
| Bb tRNA^Met^ 3' | tggTGCTGATGGAGGGACTTGAACCCACGACAACTCGGATATGAGCCGAG |

*Burkholderia cenocepacia*

| BcTilS lift primers |  |
| --- | --- |
| BcTilS Lift forward | CGCTGCGCCAGTTCCAGGGCATGAGCATCGATGCGCTGC |
| BcTilS Lift reverse | GCGTGGCGGATTTTGCCCGGAAGGCCGATCAGGCCGCGC |
|  |  |
| BcTilS cloning |  |
| BcTilS NheI forward | AAAAAAGCTAGCGTGATCCCACCCACCGAATTCTCCGC |
| BcTilS HindIII reverse | AAAAAAAAGCTTCTAGGCGATCAGCAAATCGGGGCGCC |
|  |  |
| BcTilS His tag switch |  |
| remove internal NcoI forward | GATGGGCGAACGCCACGGCGTGGC |
| remove internal NcoI reverse | GCCACGCCGTGGCGTTCGCCCATC |
| NheI to NcoI forward | GCGGCAGCCATATGGCTACCATGGTCCCACCCAC |
| NheI to NcoI reverse | GTGGGTGGGACCATGGTAGCCATATGGCTGCCGC |
| remove stop forward | GATTTGCTGATCGCCGCGAAGCTTGCGGCCGC |
| remove stop reverse | GCGGCCGCAAGCTTCGCGGCGATCAGCAAATC |
|  |  |
| BcMetRS lift primers |  |
| BcMetRS Lift forward | CCCGCCCGCCGCGCGAGCCCGCGCCCCTGCTAGAATCGC |
| BcMetRS Lift reverse | GGCACGTTCCAGGGCGTCGACCTGAACTACACGCGGCGG |
|  |  |
| BcMetRS cloning |  |
| BcMetRS NcoI forward | AAAAAACCATGGCCGCATCCGACCTCACTTCCGTGCAGGCTGCG |
| BcMetRS HindIII reverse | AAAAAAAAGCTTCTTCACGCGCATGCCGGGCTTCGCGCCGC |
|  |  |
| tRNA |  |
| Bc tRNA^Ile2^ sequence | GGGCCCCTAGCTCATGCTTGGTTAGAGCAGCGAACTCATAATTCGTTGGtGCCGGGTTCGACTCCCGGGGGGCCCACCA |
| Bc tRNA^Ile2^ 5' | aattcctgcagTAATACGACTCACTATAGGGCCCCTAGCTCATGCTTGGTTAGAGCAGCGAACTCATAATTC |
| Bc tRNA^Ile2^ 3' | TGGTGGGCCCCCCGGGAGTCGAACCCGGCACCAACGAATTATGAGTTCGC |
|  |  |
| Bc tRNA^Met^ sequence | GGCGAATTAGCTCAGTCGGTtAGAGCGACGGAATCATAATCCGCAGGtCCGGGGTTCGAATCCCTGATTCGCCACCA |
| Bc tRNA^Met^ 5' | aattcctgcagTAATACGACTCACTATAGGCGAATTAGCTCAGTCGGTtAGAGCGACGGAATCATAATCCGC |
| Bc tRNA^Met^ 3' | TGGTGGCGAATCAGGGATTCGAACCCCGGACCTGCGGATTATGATTCCGT |
|  |  |
| Quikchange primers |  |
| BcTilS A244T forward | CGAGCTCGGTGAGATCGTCGAGCAGCCGC |
| BcTilS A244T reverse | CGATCTGACCGAGCTCGATTTCGCCGTCG |
| BcTilS A244S forward | GCTCGACGATCTGTCCGAGCTCGATTTCGC |
| BcTilS A244S reverse | GCGAAATCGAGCTCGGACAGATCGTCGAGC |
| Bc TilS N274Y forward | CACGCGCGGCGCGTATCTGCTGCGCTTCT |
| Bc TilS N274Y reverse | AGAAGCGCAGCAGATACGCGCCGCGCGTG |
| BcTilS N274A forward | CGCGCGGCGCGGCCCTGCTGCGCTTCTGG |
| BcTilS N274A reverse | CCAGAAGCGCAGCAGGGCCGCGCCGCGCG |
| BcTilS P421L forward | CCACGCCAGCACCCCGCGCTCC |
| BcTilS P421L reverse | GGTGCTGGCCTGGCAACGCG |
| BcTilS P421A forward | GAGCGCGGGGTGGCGGCCTGGCAACGCGAC |
| BcTilS P421A reverse | GTCGCGTTGCCAGGGCCGCCACCCCGCGCTC |
| BcTilS N445K forward | GATGTCGCGCTTGACGCCCAGTC |
| BcTilS N445K reverse | GGCGTCAAGCGCGACATCGGCG |
| BcTilS N445A forward | CGCGACTGGGCGTCGCCCGCGACATCGGCG |
| BcTilS N445A reverse | CGCCGATGTCGCGGGCGACGCCCAGTCGCG |

*Bacteroides fragilis*

| BfTilS lift primers |  |
| --- | --- |
| BfTilS lift forward | ggggcatgcaaattatttagattgtttttaaataagg |
| BfTilS lift reverse | ggccagatcaatcatatattttcaaggtttatattcacaaagatacaatttatcc |
|  |  |
| BfTilS Cloning primers |  |
| BfTilS NheI forward | AAAAAAGCTAGCATGATACAAAATAGAGTAGCACAATACATCG |
| BfTilS XhoI reverse | AAAAAACTCGAGTTATTCCTCAATAAAATCTGAATGTTCGGAAACG |
|  |  |
| BfTilS His-tag switch |  |
| NheI to NcoI | gcggcagccatatggctaccatggtacaaaatagagtagca |
| remove stop | tccgaacattcagattttattgaggaagcactcgagcaccacca |
|  |  |
| tRNA primers |  |
| Bf tRNA^Ile2^ sequence | GGGCTTATAGCTCAGTTGGTTAGAGCAACAGACTCATAATCTGGAGGTCCTAGGTTCAAGCCCTAGTTGGCCCACCA |
| Bf tRNA^Ile2^ 5' | aattcctgcagTAATACGACTCACTATAGGGCTTATAGCTCAGTTGGTTAGAGCAACAGACTCATAATCTGG |
| Bf tRNA^Ile2^ 3' | TGGTGGGCCAACTAGGGCTTGAACCTAGGACCTCCAGATTATGAGTCTGT |
|  |  |
| Bf tRNA^Met^ sequence | GGCGGGATAGCTCAGCTGGTtAGAGCGCATGATTCATAATCATGAGGtCCCCGGTTCAATCCCGGGTCCCGCTACCA |
| Bf tRNA^Met^ 5' | aattcctgcagTAATACGACTCACTATAGGCGGGATAGCTCAGCTGGTtAGAGCGCATGATTCATAATCATG |
| Bf tRNA^Met^ 3' | TGGTAGCGGGACCCGGGATTGAACCGGGGaCCTCATGATTATGAATCATG |
|  |  |
| Quikchange primers |  |
| BfTilS I232T forward | GCAGCAACTTTATACAATCAAAGTACGGGGGAGGCGCGTA |
| BfTilS I232T reverse | TACGCGCCTCCCCCGTACTTTGATTGTATAAAGTTGCTGC |
| BfTilS I232A forward | CGCAGCAACTTTATACAATCAAAGTGCAGGGGAGGCGCGT |
| BfTilS I232A reverse | ACGCGCCTCCCCTGCACTTTGATTGTATAAAGTTGCTGCG |
| BfTilS E262Y forward | GGTACCCGAAGCCATTTTATTCTATGTATTACACCCGCTAGG |
| BfTilS E262Y reverse | CCTAGCGGGTGTAATACATAGAATAAAATGGCTTCGGGTACC |
| BfTilS E262A forward | CCGAAGCCATTTTATTCGCAGTATTACACCCGCTAGG |
| BfTilS E262A reverse | CCTAGCGGGTGTAATACTGCGAATAAAATGGCTTCGG |
| BfTilS S383L forward | GACTACCTGACGGATAAAAAATTCTTATTGAGTGAAAAAGAAAAGCAATGG |
| BfTilS S383L reverse | CCATTGCTTTTCTTTTTCACTCAATAAGAATTTTTTATCCGTCAGGTAGTC |
| BfTilS S383A forward | ACTACCTGACGGATAAAAAATTCGCTTTGAGTGAAAAAGAAAAGCA |
| BfTilS S383A reverse | TGCTTTTCTTTTTCACTCAAAGCGAATTTTTTATCCGTCAGGTAGT |
| BfTilS D408K forward | GGCTGATAGGAGAACGTACGAAGAACCGATTTAAGGTAAACGA |
| BfTilS D408K reverse | TCGTTTACCTTAAATCGGTTCTTCGTACGTTCTCCTATCAGCC |
| BfTilS D408A forward | CTGATAGGAGAACGTACGGCTAACCGATTTAAGGTAAAC |
| BfTilS D408A reverse | GTTTACCTTAAATCGGTTAGCCGTACGTTCTCCTATCAG |

*Escherichia coli*

| EcTilS lift primers |  |
| --- | --- |
| EcTilS lift forward | CATGACACTCACGCTCAATAGACAACTTCTCACC |
| EcTilS lift reverse | CTTAACTAAGCGTTTTCTGCCAGACAAAACTTACG |
|  |  |
| EcTilS cloning primers |  |
| EcTilS NcoI forward | ATATATACCATGGCACTCACGCTCAATAGACAACTTC |
| EcTilS XhoI reverse | AAAAAACTCGAGCTAACTAAGCGTTTTCTGCCAGACAAAAC |
|  |  |
| EcTilS His Tag switch |  |
| Nhe to Nco | ggcagccatatggctaccatggcactcacgctcaat |
| remove stop | ttttgtctggcagaaaacgcttagtgcactcgagcaccac |
|  |  |
| tRNA primers |  |
| Ec tRNA^Ile2^ sequence | GGCCCCTTAGCTCAGTGGTtAGAGCAGGCGACTCATAATCGCTTGGtCGCTGGTTCAAGTCCAGCAGGGGCCACCA |
| Ec tRNA^Ile2^ 5' | AATTCCTGCAGTAATACGACTCACTATAGGCCCCTTAGCTCAGTGGTTAGAGCAGGCGACTCATAATCGCTT |
| Ec tRNA^Ile2^ 3' | TGGTGGCCCCTGCTGGACTTGAACCAGCGACCAAGCGATTATGAGTC |
|  |  |
| Ec tRNA^Met^ sequence | GGCTACGTAGCTCAGTTGGTtAGAGCACATCACTCATAATGATGGGGtCACAGGTTCGAATCCCGTCGTAGCCACCA |
| Ec tRNA^Met^ 5' | aattcctgcagTAATACGACTCACTATAGGCTACGTAGCTCAGTTGGTtAGAGCACATCACTCATAATGATG |
| Ec tRNA^Met^ 3' | TGGTGGCTACGACGGGATTCGAACCTGTGaCCCCATCATTATGAGTGATG |
|  |  |
| Quikchange primers |  |
| EcTilS L227T forward | CTGGATGAAACGCTGGCAGATGATTTAG |
| EcTilS L227T reverse | CTAAATCATCTGCCAGCGTTTCATCCAG |
| EcTilS L227A forward | GCTGGATGAACTGGCGGCAGATGATTTAGC |
| EcTilS L227A reverse | GCTAAATCATCTGCCGCCAGTTCATCCAGC |
| EcTilS A256Y forward | GCCCGCCGCGCGTACATTATCCGCCG |
| EcTilS A256Y reverse | CGGCGGATAATGTACGCGCGGCGGGC |
| EcTilS A256S forward | GCCCGCCGCGCGTCGATTATCCGCCGCTGGC |
| EcTilS A256S reverse | GCCAGCGGCGGATAATCGACGCGCGGCGGGC |
| EcTilS P387L forward | GAGCTGGGCGTGCTGCCGTGGCTAC |
| EcTilS P387L reverse | GTAGCCACGGCAGCACGCCCAGCTC |
| EcTilS P387A forward | GGCAAGAGCTGGGCGTGGCGCCGTGGCTACG |
| EcTilS P387A reverse | CGTAGCCACGGCGCCACGCCCAGCTCTTGCC |
| EcTilS T412K forward | CAGGGGTATTTGTGAAGCAAGAAGGTGTG |
| EcTilS T412K reverse | CACACCTTCTTGCTTCACAAATACCCCTG |
| EcTilS T412A forward | GGCAGGGGTATTTGTGGCGCAAGAAGGTGTGG |
| EcTilS T412A reverse | CCACACCTTCTTGCGCCACAAATACCCCTGCC |
|  |  |

*Geobacillus kaustophilus*

| GkTilS Lift primers |  |
| --- | --- |
| GkTilS Lift forward | AAAAAAGCTAGCatgattgacaaagttcgcgccttcatccac |
| GkTilS Lift reverse | TTTTTTCTCGAGactgttcatggcttggtactggagcagaat |
|  |  |
| GkTilS cloning primers |  |
| GkTilS Nhe forward | AAAAAACCATGGGCATGATTGACAAAGTTCGCGCCTTCATCC |
| GkTilS C-term Stop | TTTTTTCTCGAGTCAACTGTTCATGGCTTGGTACTGGAGCAG |
|  |  |
| GkTilS His tag switch |  |
| GkTilS NheI to NcoI forward | gccatatggctaCCATGGttgacaaagttc |
| GkTilS NheI to NcoI reverse | gaactttgtcaaCCATGGtagccatatggc |
|  |  |
| Gk tRNA^Ile2^ sequence | GGACCTTTAGCTCAGCTGGTtAGAGCAGACGGCTCATAACCGTCCGGTCGTAGGTTCGAGTCCTACAAGGTCCACCA |
| Gk tRNA^Ile2^ 5′ | aattcctgcagTAATACGACTCACTATAGGACCTTTAGCTCAGCTGGTtAGAGCAGACGGCTCATAACCGTC |
| Gk tRNA^Ile2^ 3′ | TGGTGGACCTTGTAGGACTCGAACCTACGACCGGACGGTTATGAGCCGTC |
|  |  |
| Gk tRNA^Met^ sequence | GGCGGTGTAGCTCAGCTGGCtAGAGCGTACGGTTCATACCCGTGAGGtCGGGGGTTCGATCCCCTCCACCGCCACCA |
| Gk tRNA^Met^ 5′ | aattcctgcagTAATACGACTCACTATAGGCGGTGTAGCTCAGCTGGCtAGAGCGTACGGTTCATACCCGTG |
| Gk tRNA^Met^ 3′ | TGGTGGCGGTGGAGGGGATCGAACCCCCGaCCTCACGGGTATGAACCGTA |
|  |  |
| Quikchange primers |  |
| GkTilS A239T forward | GAGCAATTTTTGGAGGAATTAACCGCGGACGCG |
| GkTilS A239T reverse | CGCGTCCGCGGTTAATTCCTCCAAAAATTGCTC |
| GkTilS A239S forward | AGATGAGCAATTTTTGGAGGAATTAAGCGCGGACGCGCTG |
| GkTilS A239S reverse | CAGCGCGTCCGCGCTTAATTCCTCCAAAAATTGCTCATCT |
| GkTilS Q274Y forward | AGCGCCGGGTGCTGTATCTGTTGCTTCTCCG |
| GkTilS Q274Y reverse | CGGAGAAGCAACAGATACAGCACCCGGCGCT |
| GkTilS Q274A forward | GCGCCGGGTGCTGGCGCTGTTGCTTCTC |
| GkTilS Q274A reverse | GAGAAGCAACAGCGCCAGCACCCGGCGC |
| GkTilS P416L forward | GAAATTTTCATTGAAGCAAAAATTCTGCGGATGGAAAGGGATC |
| GkTilS P416L reverse | GATCCCTTTCCATCCGCAGAATTTTTGCTTCAATGAAAATTTC |
| GkTilS P416A forward | TGAAAGAAATTTTCATTGAAGCAAAAATTGCGCGGATGGAAAGGG |
| GkTilS P416A reverse | CCCTTTCCATCCGCGCAATTTTTGCTTCAATGAAAATTTCTTTCA |
| GkTilS S442K forward | CCTTTGGGTGCCTGGCTTGAAAAAAAAGGCTTTCGAAGCG |
| GkTilS S442K reverse | CGCTTCGAAAGCCTTTTTTTTCAAGCCAGGCACCCAAAGG |
| GkTilS S442A forward | TTTGGGTGCCTGGCTTGAAAAAAGCGGCTTTCGAAG |
| GkTilS S442A reverse | CTTCGAAAGCCGCTTTTTTCAAGCCAGGCACCCAAA |

*Helicobacter pylori*

| Lift primers |  |
| --- | --- |
| HpTilS lift forward | ggcgttttaacccacaaggaaacttcataaaagcgc |
| HpTilS lift reverse | gccgtattttgacacaatcaaggttaaaaagcgc |
|  |  |
| Cloning primers |  |
| HpTilS NheI forward | AAAAAAGCTAGCGTGCAAGATTTTAAAACCCATTTAGAGCC |
| HpTilS XhoI reverse | AAAAAACTCGAGTTATAGATCCATTAAACTCTGTTTGAAACGGG |
|  |  |
| His tag switch |  |
| HpTilS NheI to NcoI forward | CGCGGCAGCCATATGGCTACCATGGAAGATTTTAAAACCCATTTA |
| HpTilS NheI to NcoI reverse | TAAATGGGTTTTAAAATCTTCCATGGTAGCCATATGGCTGCCGCG |
| HpTilS remove stop forward | CCGTTTCAAACAGAGTTTAATGGATCTAGCACTCGAGCACCACCAC |
| HpTilS remove stop reverse | GTGGTGGTGCTCGAGTGCTAGATCCATTAAACTCTGTTTGAAACGG |
|  |  |
| tRNA primers |  |
| Hp tRNA^Ile2^ sequence | GGATTCTTAGCTCAGCTGGTCAGAGCACTCGGCTCATAACCGATTGGTCGTAGGTTCAAGTCCTACAGAATCCACCACCA |
| Hp tRNA^Ile2^ 5' primer | aattcctgcagTAATACGACTCACTATAGGATTCTTAGCTCAGCTGGTCAGAGCACTCGGCTCATAACCGAT |
| Hp tRNA^Ile2^ 3' primer | TGGTGGTGGATTCTGTAGGACTTGAACCTACGACCAATCGGTTATGAGCCGAG |
|  |  |
| Hp tRNA^Met^ sequence | GTCAAGGTAGCTCAGCTGGTttAGAGCGCTGGTCTCATAAGCCGGAGGtCGGGGGTTCAAGTCCCCCTCTTGACACCA |
| Hp tRNA^Met^ 5' primer | aattcctgcagTAATACGACTCACTATAGTCAAGGTAGCTCAGCTGGTttAGAGCGCTGGTCTCATAAGCCG |
| Hp tRNA^Met^ 3' primer | TGGTGTCAAGAGGGGGACTTGAACCCCCGaCCTCCGGCTTATGAGACCAGC |

*Mycoplasma penetrans*

| Lift primers |  |
| --- | --- |
| MpTilS forward | gcgctgaagatagtattaaagttcaacaagcaattattaaagcaaagg |
| MpTilS reverse | ccttttcaataatttctaatgatgaaaatccaatatttccagttgctcc |
|  |  |
| Cloning primers |  |
| MpTilS NheI forward | GGTGGTGCTAGCATGAAACATAAGTATTTAATTGCTGTTTCTGG |
| MpTilS XhoI reverse | GGTGGTCTCGAGCTAACAAATAGAATATAGTATTTTTTTATTTTTGATTTCAATAAATTG |
|  |  |
| Stop to Trp primers |  |
| MpTilS X80W forward | GTATCTAAATACAAAGATATTAATAATTTACAAACTTGGTATAGAGAAATAAGATATGATTTTTTTGAGAAAATATC |
| MpTilS X80W reverse | GATATTTTCTCAAAAAAATCATATCTTATTTCTCTATACCAAGTTTGTAAATTATTAATATCTTTGTATTTAGATAC |
| MpTilS X171W forward | TATAGAATTTGTAATAGACTATACAAATTTTTGGGATAGATATAGTAGAAATGTTGTTAGAAAAATG |
| MpTilS X171W reverse | CATTTTTCTAACAACATTTCTACTATATCTATCCCAAAAATTTGTATAGTCTATTACAAATTCTATA |
| MpTilS X186W forward | GAAATGTTGTTAGAAAAATGATGGCAGAATGGGATAAAAAAACTTTTCAAAAATTTTATCTTAA |
| MpTilS X186W reverse | TTAAGATAAAATTTTTGAAAAGTTTTTTTATCCCATTCTGCCATCATTTTTCTAACAACATTTC |
| MpTilS X200W forward | AAAACTTTTCAAAAATTTTATCTTAAGGTCAAATGGTTCAATCTGAAAAATATGTTTTTCATAAAACTATT |
| MpTilS X200W reverse | AATAGTTTTATGAAAAACATATTTTTCAGATTGAACCATTTGACCTTAAGATAAAATTTTTGAAAAGTTTT |
| MpTilS X219W forward | ATGTTTTTCATAAAACTATTGGATTCTAAATTTAATAATTGGATCAAACAGGATTTTGATATTAATTATTTTTTAAAAATA |
| MpTilS X219W reverse | TATTTTTAAAAAATAATTAATATCAAAATCCTGTTTGATCCAATTATTAAATTTAGAATCCAATAGTTTTATGAAAAACAT |
|  |  |
| His tag switch |  |
| MpTilS NheI to NcoI forward | CGGCAGCCATATGGCTACCATGGAGCACAAGTATCTG |
| MpTilS NheI to NcoI reverse | CAGATACTTGTGCTCCATGGTAGCCATATGGCTGCCG |
| MpTilS remove stop forward | AATTCTGTATAGTATCTGCGCACTCGAGCACCACCACCAC |
| MpTilS remove stop reverse | GTGGTGGTGGTGCTCGAGTGCGCAGATACTATACAGAATT |
|  |  |
| tRNA primers |  |
| Mp tRNA^Ile2^ sequence | GGAGCTATAGCTCAACTGGTTAGAGCCCCCGACTCATAATCGGTAGGTTACAGGTTCAAGTCCTGTTAGCTCCACCA |
| Mp tRNA^Ile2^ 5' primer | aattcctgcagTAATACGACTCACTATAGGAGCTATAGCTCAACTGGTTAGAGCCCCCGACTCATAATCGGT |
| Mp tRNA^Ile2^ 3' primer | CCCGACTCATAATCGGTAGGTTACAGGTTCAAGTCCTGTTAGCTCCACCA |
|  |  |
| Mp tRNA^Met^ sequence | GGCAGAGTATCTCAGTGGTtAGAGAACTCGGCTCATACCCGAGGTGtCGAGAGTTCGAATCTCTCCTCTGTCACCA |
| Mp tRNA^Met^ 5' primer | AATTCCTGCAGTAATACGACTCACTATAGGCAGAGTATCTCAGTGGTTAGAGAACTCGGCTCATACCCGAGG |
| Mp tRNA^Met^ 3' primer | TGGTGACAGAGGAGAGATTCGAACTCTCGACACCTCGGGTATGAGCCGAG |
|  |  |

*Mycobacterium smegmatis*

| Lift primers |  |
| --- | --- |
| MsTilS lift forward | ccgcggtggggatgcaacggttcaacaccgtgtggacgaacgccg |
| MsTilS lift reverse | cccggatacatctcggtggattcgacagccacgtccacagcgtgcc |
|  |  |
| Cloning primers |  |
| NheI forward | AAAAAAGCTAGCATGGATCGATCGCGTGCTGTAGCGACGCTGCG |
| XhoI reverse | AAAAAACTCGAGTCACACCGGTTCGGTGTAGAGCACCAACTCGC |
|  |  |
| MsTilS His tag switch |  |
| NheI to NcoI | gcagccatatggctaccatggatcgatcgcg |
| XhoI to stop | acaccgaaccggtggcactcgagcaccacc |
|  |  |
| tRNA primers |  |
| Ms tRNA^Ile2^ sequence | GGGGCGGTAGCTCAGTCGGTTAGAGCCGCGGACTCATAATCCGCTGGTCGCGGGTTCGAGCCCCGCCCGCCCTACCA |
| Ms tRNA^Ile2^ 5' | aattcctgcagTAATACGACTCACTATAGGGGCGGTAGCTCAGTCGGTTAGAGCCGCGGACTCATAATCCGC |
| Ms tRNA^Ile2^ 3' | TGGTAGGGCGGGCGGGGCTCGAACCCGCGACCAGCGGATTATGAGTCCGC |
|  |  |
| Ms tRNA^Met^ sequence | GGCGGTGTAGCTCAGCTGGTtAGAGCGCACGACTCATAATCGTGAGGtCGGGGGATCGAGCCCCCCCACCGCTAcca |
| Ms tRNA^Met^ 5' | aattcctgcagTAATACGACTCACTATAGGCGGTGTAGCTCAGCTGGTtAGAGCGCACGACTCATAATCGTG |
| Ms tRNA^Met^ 3' | tggTAGCGGTGGGGGGGCTCGATCCCCCGaCCTCACGATTATGAGTCGTG |
|  |  |
| Quikchange primers |  |
| MsTilS A228T forward | CTCGACGAGATCACGAGGCAGGCCC |
| MsTilS A228T reverse | GGGCCTGCCTCGTGATCTCGTCGAG |
| MsTilS A228S forward | CTCGACGAGATCTCGAGGCAGGCCC |
| MsTilS A228S reverse | GGGCCTGCCTCGAGATCTCGTCGAG |
| MsTilS R260Y forward | GTGCCGTGCGGCGCTATGTGATCCGGGCCTG |
| MsTilS R260Y reverse | CAGGCCCGGATCACATAGCGCCGCACGGCAC |
| MsTilS R260A forward | CCGTGCGGCGCGCCGTGATCCGGG |
| MsTilS R260A reverse | CCCGGATCACGGCGCGCCGCACGG |
| MsTilS R153A forward | CCGCCGTGGCACGCGCCGCTTCTGGG |
| MsTilS R153A reverse | CCCAGAAGCGGCGCGTGCCACGGCGG |
| MsTilS R191A forward | CAGGACCCGGCTGGCCCTCGATGTGATG |
| MsTilS R191A reverse | CATCACATCGAGGGCCAGCCGGGTCCTG |
| MsTilS D35A forward | CGGTGGGCCGGCCTCGCTTGCGC |
| MsTilS D35A reverse | GCGCAAGCGAGGCCGGCCCACCG |
| MsTilS E126A forward | GACGATCAGGCCGCGACCGTGCTGCTG |
| MsTilS E126A reverse | CAGCAGCACGGTCGCGGCCTGATCGTC |

*Pseudomonas aeruginosa*

| Lift primers |  |
| --- | --- |
| PaTilS lift forward | GACCGCCTGATGAGCTACGGCGCGCCGTAAGCCATC |
| PaTilS lift reverse | CCCGCCACGGACAAATCCGTGGGCAAAGCAAACTCCTGCTG |
|  |  |
| Cloning primers |  |
| PaTilS NheI forward | AAAAAAGCTAGCATGCCGGCGGTGCCGCTCGAATCCCGTCTGTT |
| PaTilS XhoI reverse | AAAAAACTCGAGCTAGCTCAAACCCGGGTCGTTCGTCGGCGGGC |
|  |  |
| PaTilS His tag switch |  |
| PaTilS NheI to NcoI | gcagccatatggctaccatggcggcggtg |
| PaTilS XhoI to stop | acccgggtttgagcgcgaagcttgcggccg |
|  |  |
| Lift primers |  |
| PaMetRS lift forward | GATGCCGGTCATCCTACCCGAGTGGCGCGCGGATGAAAAAAAACC |
| PaMetRS lift reverse | GTCTCGCCCTGGAAACCGGCATTGCCGGAGACGCTTTC |
|  |  |
| Cloning primers |  |
| PaMetRS NheI forward | AAAAAAGCTAGCATGTCCGAACCACGCAAGATCCTCGTTACCAGCGCCCTCC |
| PaMetRS HindIII reverse | AAAAAAAAGCTTTTACTTGACGCGCTGGCCCGGCTTGGCGCCGC |
|  |  |
| tRNA primers |  |
| Pa tRNA^Ile2^ sequence | GGGCCTATAGCTCAGTCGGTTAGAGCAGAGGACTCATAATCCTTTGGTCCACGGTTCGAGTCCGTGTGGGCCCACCA |
| Pa tRNA^Ile2^ 5' | AATTCCTGCAGTAATACGACTCACTATAGGGCCTATAGCTCAGTCGGTtAGAGCAGAGGACTCATAATCCTT |
| Pa tRNA^Ile2^ 3' | TGGTGGGCCCACACGGACTCGAACCGTGGaCCAAAGGATTATGAGTCCTC |
|  |  |
| Pa tRNA^Met^ sequence | GGCTACATAGCTCAGTCGGTTAGAGCGCAGCATTCATAATGCTGATGTCCCAGGTTCAAGTCCCGGTGTAGCCACCA |
| Pa tRNA^Met^ 5' | aattcctgcagTAATACGACTCACTATAGGCTACATAGCTCAGTCGGTTAGAGCGCAGCATTCATAATGCTG |
| Pa tRNA^Met^ 3' | TGGTGGCTACACCGGGACTTGAACCTGGGACATCAGCATTATGAATGCTG |
|  |  |

*Staphylococcus pneumoniae*

| Lift primers |  |
| --- | --- |
| SpTilS lift forward | ggagatgcggatgaatttaagcatgatacg |
| SpTilS lift reverse | ccaccatggtagctagaaaccatcatgaagtc |
|  |  |
| Cloning primers |  |
| SpTilS NheI forward | AAAAAAGCTAGCATGAGGGAACCAGATTTTTTAAATCATTTTCTC |
| SpTilS XhoI reverse | AAAAAACTCGAGTTACCTATCTATTTTTTCTATATAAAGTACAG |
|  |  |
| SpTilS His tag switch |  |
| SpTilS NheI to NcoI forward | GCAGCCATATGGCTACCATGGGTGAACCGGACTT |
| SpTilS NheI to NcoI reverse | AAGTCCGGTTCACCCATGGTAGCCATATGGCTGC |
| SpTilS remove stop forward | TCGAGAAAATCGATCGCGCACTCGAGCACCACCACC |
| SpTilS remove stop reverse | GGTGGTGGTGCTCGAGTGCGCGATCGATTTTCTCGA |
|  |  |
| tRNA primers |  |
| Sp tRNA^Ile2^ sequence | GGACCTTTAGCTCAGCTGGTTAGAGCTCTCGGCTCATAACCGAGTGGTCGTAGGTTCAAGTCCTACAAGGTCCACCA |
| Sp tRNA^Ile2^ 5' | aattcctgcagTAATACGACTCACTATAGGACCTTTAGCTCAGCTGGTTAGAGCTCTCGGCTCATAACCGAG |
| Sp tRNA^Ile2^ 3' | TGGTGGACCTTGTAGGACTTGAACCTACGACCACTCGGTTATGAGCCGAG |
|  |  |
| Sp tRNA^Met^ sequence | GGCGGTGTAGCTCAGCTGGCtAGAGCGTCCGGTTCATACCCGGGAGGTCGGGGGTTCGATCCCCTTCGCCGCTAcca |
| Sp tRNA^Met^ 5' | aattcctgcagTAATACGACTCACTATAGGCGGTGTAGCTCAGCTGGCTAGAGCGTCCGGTTCATACCCGGG |
| Sp tRNA^Met^ 3' | tggTAGCGGCGAAGGGGATCGAACCCCCGACCTCCCGGGTATGAACCGGA |

| Bc tRNA^Ile2^ sequence | GGGCCCCTAGCTCATGCTTGGTTAGAGCAGCGAACTCATAATTCGTTGGTGCCGGGTTCGACTCCCGGGGGGCCCACCA |
| --- | --- |
| Bc tRNA^Ile2^ 5' | aattcctgcagTAATACGACTCACTATAGGGCCCCTAGCTCATGCTTGGTTAGAGCAGCGAACTCATAATTC |
| Bc tRNA^Ile2^ 3' | mUmGGTGGGCCCCCCGGGAGTCGAACCCGGCACCAACGAATTATGAGTTCGC |
|  |  |
| Ec tRNA^Ile2^ sequence | GGCCCCTTAGCTCAGTGGTTAGAGCAGGCGACTCATAATCGCTTGGTCGCTGGTTCAAGTCCAGCAGGGGCCACCA |
| Ec tRNA^Ile2^ 5' | AATTCCTGCAGTAATACGACTCACTATAGGCCCCTTAGCTCAGTGGTTAGAGCAGGCGACTCATAATCGCTT |
| Ec tRNA^Ile2^ 3' | mUmGGTGGCCCCTGCTGGACTTGAACCAGCGACCAAGCGATTATGAGTC |
|  |  |
| tRNA #3 chimera | GGGCCCCTAGCTCATGCTTGGTtAGAGCAGCGAACTCATAATTCGTTGGTGCCGGGTTCGACTCCCGGGGGGCCCACCA |
| tRNA#3 5' (same as wild-type) | AATTCCTGCAGTAATACGACTCACTATAGGCCCCTTAGCTCAGTGGTTAGAGCAGGCGACTCATAATCGCTT |
| tRNA#3 3' | mUmGGTGGCCCCTGCGGGACTTGAACCCGCGACCAAGCGATTATGAGTC |
|  |  |
| tRNA #4 chimera (G51:C63) | GGCCCCTTAGCTCAGTGGTTAGAGCAGGCGACTCATAATCGCTTGGTCGCGGGTTCAAGTCCCGCAGGGGCCACCA |
| tRNA#4 5' | AATTCCTGCAGTAATACGACTCACTATAGGCCCCTTAGCTCAGTGGTTAGAGCAGCGAACTCATAATTCGTT |
| tRNA#4 3' | mUmGGTGGCCCCTGCTGGACTTGAACCAGCGACCAACGAATTAT |
|  |  |
| tRNA #5 chimera (G3:C70) | GGGCCCTTAGCTCAGTGGTTAGAGCAGGCGACTCATAATCGCTTGGTCGCTGGTTCAAGTCCAGCAGGGCCCACCA |
| tRNA#5 5' | AATTCCTGCAGTAATACGACTCACTATAGGGCCCTTAGCTCAGTGGTTAGAGCAGGCGACTCATAATCGCTT |
| tRNA#5 3' | mUmGGTGGGCCCTGCTGGACTTGAACCAGCGACCAAGCGATTATGAGTC |
|  |  |
| tRNA #6 chimera (D-loop) | GGCCCCTTAGCTCATGCTTGGTTAGAGCAGGCGACTCATAATCGCTTGGTCGCTGGTTCAAGTCCAGCAGGGGCCACCA |
| tRNA#6 5' | AATTCCTGCAGTAATACGACTCACTATAGGCCCCTTAGCTCATGCTTGGTTAGAGCA |
| tRNA#6 3' (same as wild-type) | mUmGGTGGCCCCTGCTGGACTTGAACCAGCGACCAAGCGATTATGAGTC |
|  |  |
| tRNA #7 chimera (D-loop + G3:C70) | GGGCCCTTAGCTCATGCTTGGTTAGAGCAGGCGACTCATAATCGCTTGGTCGCTGGTTCAAGTCCAGCAGGGCCCACCA |
| tRNA#7 5' | AATTCCTGCAGTAATACGACTCACTATAGGGCCCTTAGCTCATGCTTGGTTAGAGCA |
| tRNA#7 3' (same as tRNA#5 3') | mUmGGTGGGCCCTGCTGGACTTGAACCAGCGACCAAGCGATTATGAGTC |

Chimeric tRNAs

**References**

1. Katayama, H., McGill, M., Kearns, A., Brzozowski, M., Degner, N., Harnett, B., Kornilayev, B., Matković-Čalogović, D., Holyoak, T., Calvet, J. P., Gogol, E. P., Seed, J., and Fisher, M. T. (2009) Strategies for folding of affinity tagged proteins using GroEL and osmolytes. *J Struct Funct Genomics*. **10**, 57–66

2. Loberg, M. A., Hurtig, J. E., Graff, A. H., Allan, K. M., Buchan, J. A., Spencer, M. K., Kelly, J. E., Clodfelter, J. E., Morano, K. A., Lowther, W. T., and West, J. D. (2019) Aromatic Residues at the Dimer−Dimer Interface in the Peroxiredoxin Tsa1 Facilitate Decamer Formation and Biological Function. *Chem. Res. Toxicol.* **32**, 474–483

3. Mauney, C. H., Rogers, L. C., Harris, R. S., Daniel, L. W., Devarie-Baez, N. O., Wu, H., Furdui, C. M., Poole, L. B., Perrino, F. W., and Hollis, T. (2017) The SAMHD1 dNTP Triphosphohydrolase Is Controlled by a Redox Switch. *Antioxidants & Redox Signaling*. **27**, 1317–1331

4. Konczal, J., Bower, J., and Gray, C. H. (2019) Re-introducing non-optimal synonymous codons into codon-optimized constructs enhances soluble recovery of recombinant proteins from Escherichia coli. *PLoS ONE*. **14**, e0215892

5. Nakanishi, K., Bonnefond, L., Kimura, S., Suzuki, T., Ishitani, R., and Nureki, O. (2009) Structural basis for translational fidelity ensured by transfer RNA lysidine synthetase. *Nature*. **461**, 1144–8

6. Kuratani, M., Yoshikawa, Y., Bessho, Y., Higashijima, K., Ishii, T., Shibata, R., Takahashi, S., Yutani, K., and Yokoyama, S. (2007) Structural Basis of the Initial Binding of tRNAIle Lysidine Synthetase TilS with ATP and L-Lysine. *Structure*. **15**, 1642–1653

7. Gasteiger, E., Hoogland, C., Gattiker, A., Duvaud, S., Wilkins, M. R., Appel, R. D., and Bairoch, A. (2005) Protein Identification and Analysis Tools on the ExPASy Server. in *The Proteomics Protocols Handbook* (Walker, J. M. ed), pp. 571–607, Humana Press, Totowa, NJ, 10.1385/1-59259-890-0:571

8. Notredame, C., Higgins, D. G., and Heringa, J. (2000) T-coffee: a novel method for fast and accurate multiple sequence alignment 1 1Edited by J. Thornton. *Journal of Molecular Biology*. **302**, 205–217
